# Supplementary material for: Miniaturized chaos-assisted Spectrometer
Source: Light Sci Appl. 2025 Sep 18;14:331. doi: 10.1038/s41377-025-01984-x (PMC12446452; doi:10.1038/s41377-025-01984-x)
Supplement: Supplementary file 1 — Supplementary Information for Miniaturized Chaos-assisted Spectrometer [file 41377_2025_1984_MOESM1_ESM.docx]

﻿Supplementary Information for

**Miniaturized Chaos-assisted Spectrometer**

Yujia Zhang^1,†^, Chaojun Xu^1,†^, Zhenyu Zhao^1^, Yikai Su^1,*^ , Xuhan Guo^1,*^

^1^ State Key Laboratory of Photonics and Communications, School of Information and Electronic Engineering, Shanghai Jiao Tong University; Shanghai, 200240, China.

*Corresponding author. Email: [guoxuhan@sjtu.edu.cn](mailto:yangzongyin@zju.edu.cn); yikaisu@sjtu.edu.cn.

†These authors contributed equally to this work.

**S1. Dynamic analysis of momentum transformation**

The dynamics of the momentum transformation are explored using full three-dimensional finite-difference time-domain (3D FDTD) simulations. A short pulse of probe light with a duration of 10 fs is used to excite resonant modes. Temporal distributions of field intensity during the momentum transformation at a resonant frequency are recorded. Fig. **S1** to **S3** illustrate temporal evolution and corresponding temporal distributions of fields of some mode patterns at 1531.75 nm, 1541.350 nm, and 1507.75 nm. In Fig. **S1**, within 0.225 ps, light is injected into the chaotic sea and enters the chaotic channels. From 0.225 to 30 ps, the light dynamically tunnels into an orbital resonant mode pattern from chaotic channels. At 30 ps, about 93% of the electric-field distribution is already attributable to the corresponding stabilized mode pattern. After 30 ps, this orbital mode pattern maintains stability for its long lifetime, as evidenced by the consistent field distribution observed at both 30 ps and 200 ps. Additionally, other resonant modes also emerge a similar tendency, as exhibited in Fig. **S2** and **S3**. Therefore, the chaotic motions convert the angular momentum of light into stable orbital resonant mode patterns within a few dozen picoseconds, demonstrating excellent temporal stability for spectral matrix reconstruction. These resonant mode patterns standing for different wavelengths reveal ultrafast stabilizing characteristics in the time domain as well as depict diversity in the wavelength domain.


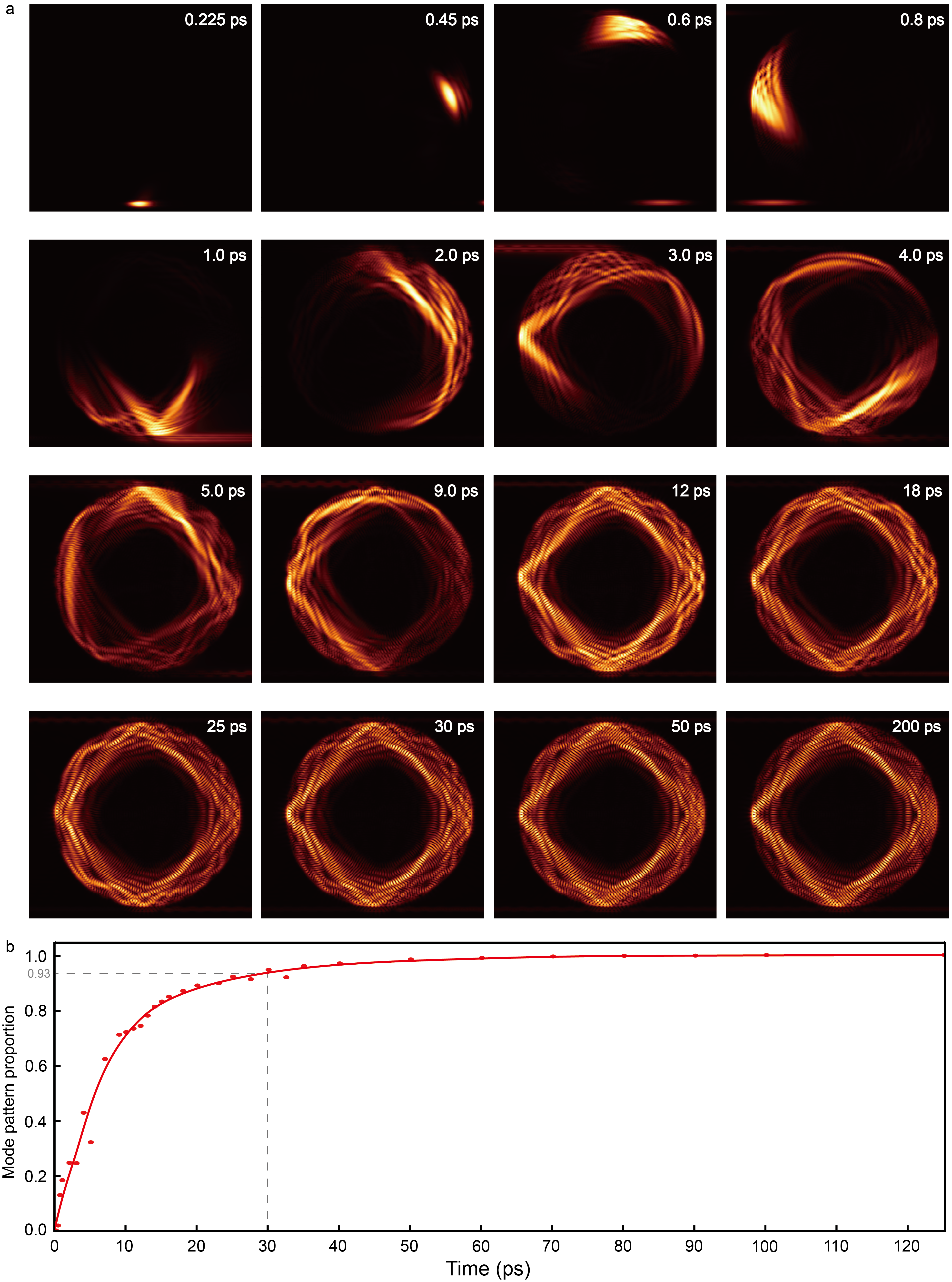


**Fig. S1 | a,** Short-time snapshots of the 3D FDTD simulation depicting field distributions at 1531.75 nm of the chaotic cavity from 0.225 ps to 200 ps. The consistency of field distributions in 30 ps and 200 ps demonstrates ultrafast resonant mode stabilizing in the time domain as well as depicting diversity in the wavelength domain. **b,** Temporal evolution of stabilized mode pattern proportion derived from the field intensity distribution. At 30 ps, about 93% of the electric-field distribution is already attributable to the corresponding stabilized mode pattern.


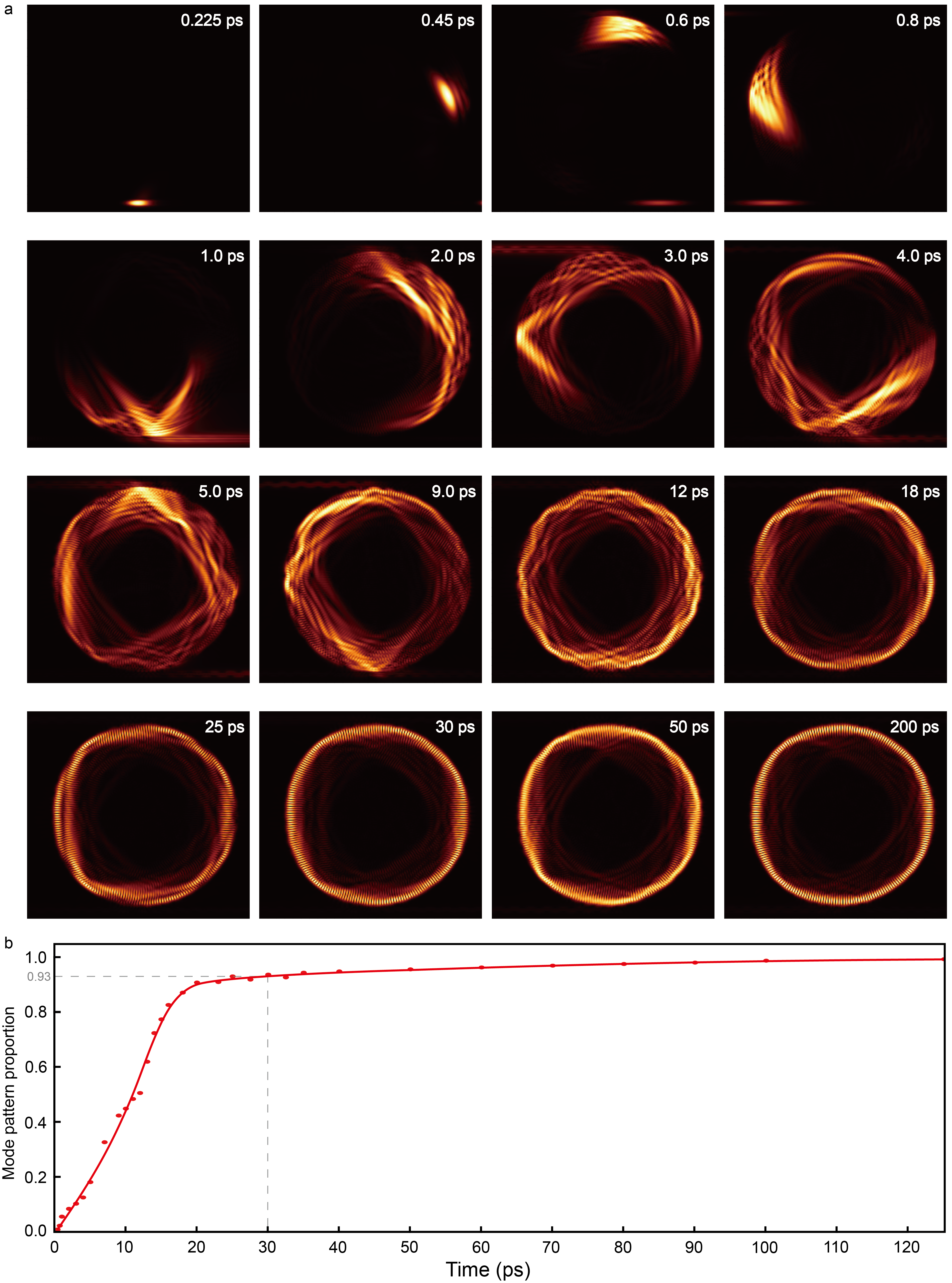


**Fig. S2 |** **a**, Short-time snapshots of the 3D FDTD simulation depicting field distributions at 1541.50 nm of the chaotic cavity from 0.225 ps to 200 ps. The consistency of field distributions in 30 ps and 200 ps demonstrates ultrafast resonant mode stabilizing in the time domain as well as depicting diversity in the wavelength domain. **b**, Temporal evolution of stabilized mode pattern proportion derived from the field intensity distribution. At 30 ps, about 93% of the electric-field distribution is already attributable to the corresponding stabilized mode pattern.


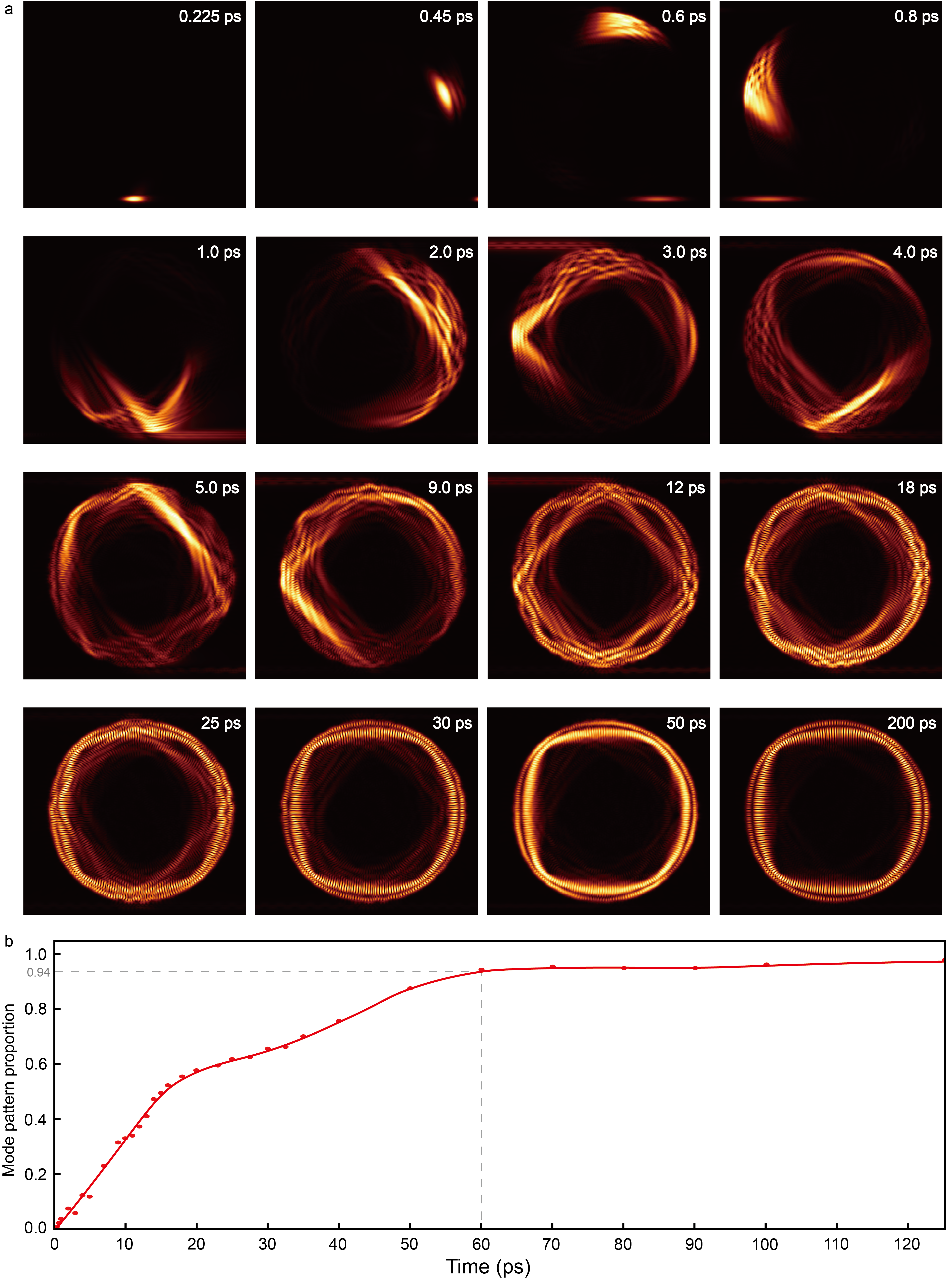


**Fig. S3 |** **a,** Short-time snapshots of the 3D FDTD simulation depicting field distributions at 1507.75 nm of the chaotic cavity from 0.225 ps to 200 ps. The consistency of field distributions in 30 ps and 200 ps demonstrates ultrafast resonant mode stabilizing in the time domain as well as depicting diversity in the wavelength domain. **b,** Temporal evolution of stabilized mode pattern proportion derived from the field intensity distribution. At 60 ps, about 94% of the electric-field distribution is already attributable to the corresponding stabilized mode pattern.

**S2. Initial deformation parameter scanning**

Exploiting the mathematical principles of the ray dynamics model and utilizing the Poincaré surface of section (PSOS), we simulate and evaluate the chaotic motion of light in cavities. Initially, we identify the coarse range of the deformation parameter *α* from 0 to 0.5, as illustrated in Fig. **S4**.


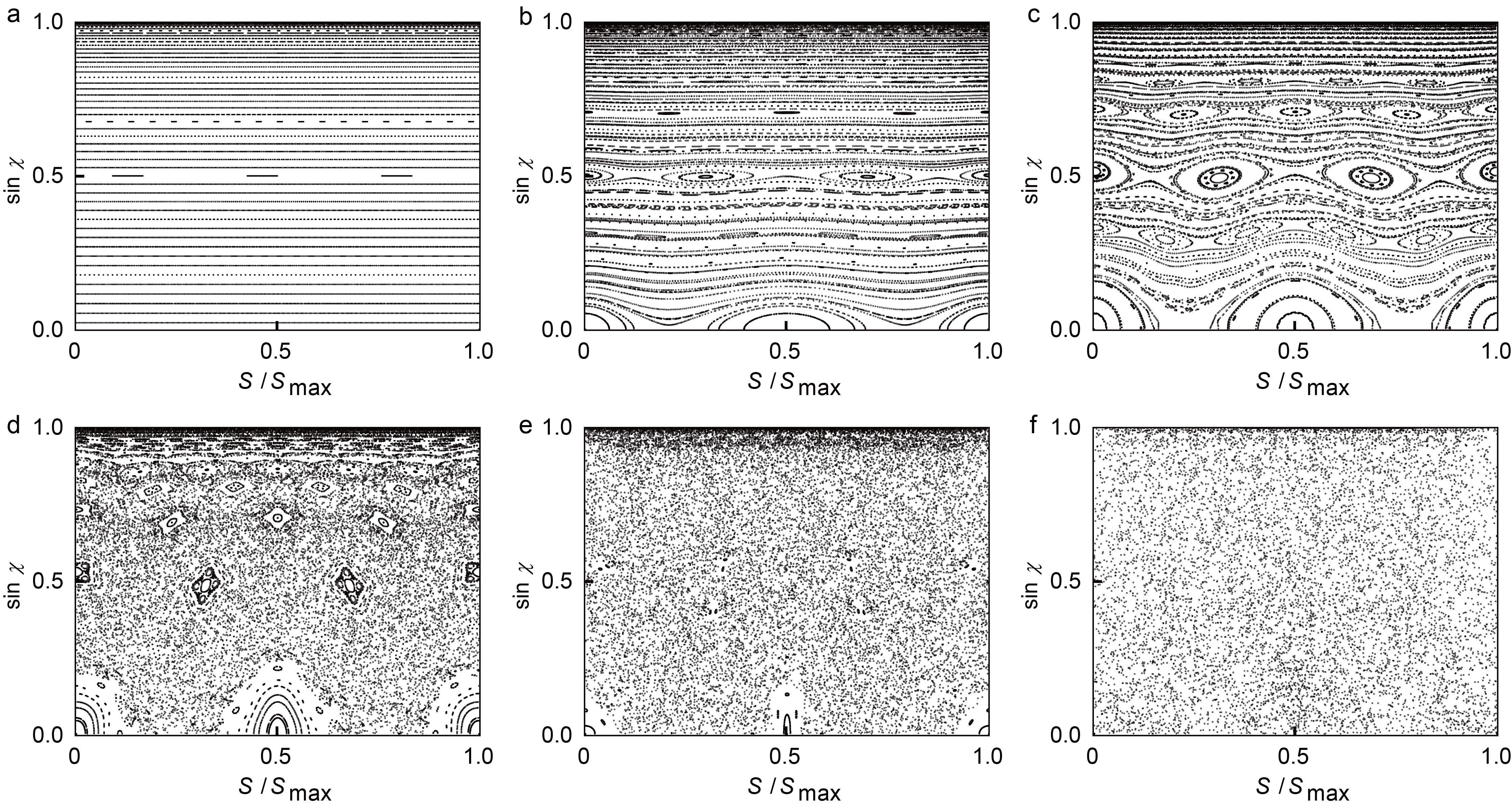


**Fig. S4 |** Calculated PSOS with different deformation parameters *α* = 0 (**a**), 0.1 (**b**), 0.2 (**c**), 0.3 (**d**), 0.4 (**e**), 0.5(**f**).

**S3. Circular microdisk transmission and auto-correlation**

We fabricated a series of chaotic cavities with different *α* (*α* = 0, 0.3, 0.35, 0.375. 0.4, 0.45, 0.5) with the same effective radius of 10 μm in the add-drop configuration, where the width of two bus waveguides is 450 nm and the gap between the bus waveguides and the chaotic cavity is 150 nm. The spectral response is measured from the drop ports by gratings and then normalized. Firstly, the transmission spectrum of the circular microdisk cavity (*α* = 0) is exhibited in Fig. **S5a**. Auto-correlation function, which is plotted in Fig. **S5b**, is computed to assess the level of decorrelation and the presence of hidden periodicity in the transmission spectrum. According to the defined F.O.M in the manuscript, the black dashed line of 0.55 indicates a prominent level of periodicity.


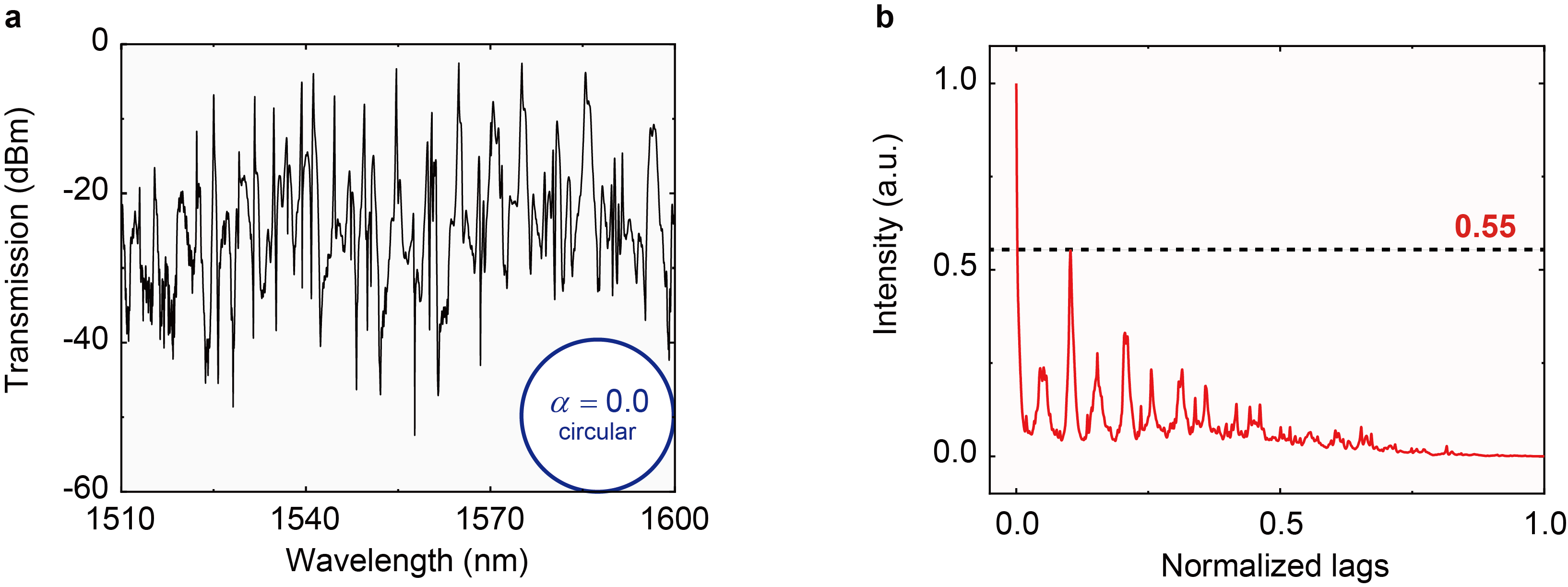


**Fig. S5 |** **a,** Measured transmission spectrum from drop port of circular cavity. **b,** Calculated auto-correlation function where black dashed line labels the F.O.M corresponding to periodicity.

**S4. Transmission and auto-correlation for chaotic cavities with different α**

The transmission spectra measured from other chaotic cavities with non-zero α and calculated corresponding auto-correlation functions are illustrated in Fig. **S6** (*α* = 0.3 (**a**), 0.35 (**b**), 0.375 (**c**), 0.4 (**d**), 0.45 (**e**) and 0.5 (**f**)). Obviously, the periodicity is gradually suppressed to about 0.23 when α increases to 0.375 and then becomes larger in turn when *α* increases to 0.4.


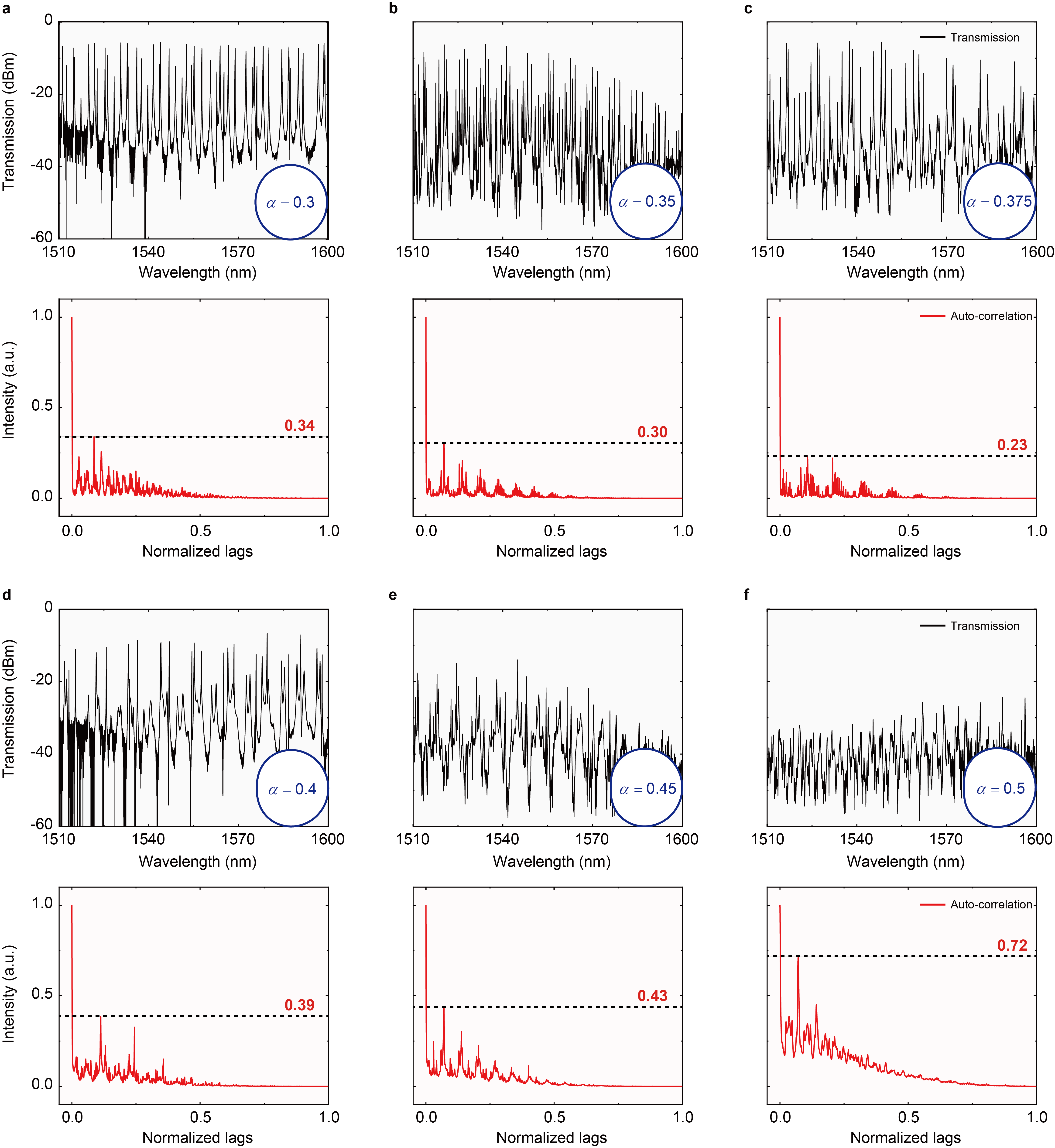


**Fig. S6 |** Measured transmission spectrum from drop port (black lines and backgrounds) and calculated auto-correlation function of these spectra (red lines and red backgrounds) when *α* = 0.3 (**a**), 0.35 (**b**), 0.375 (**c**), 0.4 (**d**), 0.45 (**e**), 0.5 (**f**).

**S5. Numerically calculated electric-field distributions**


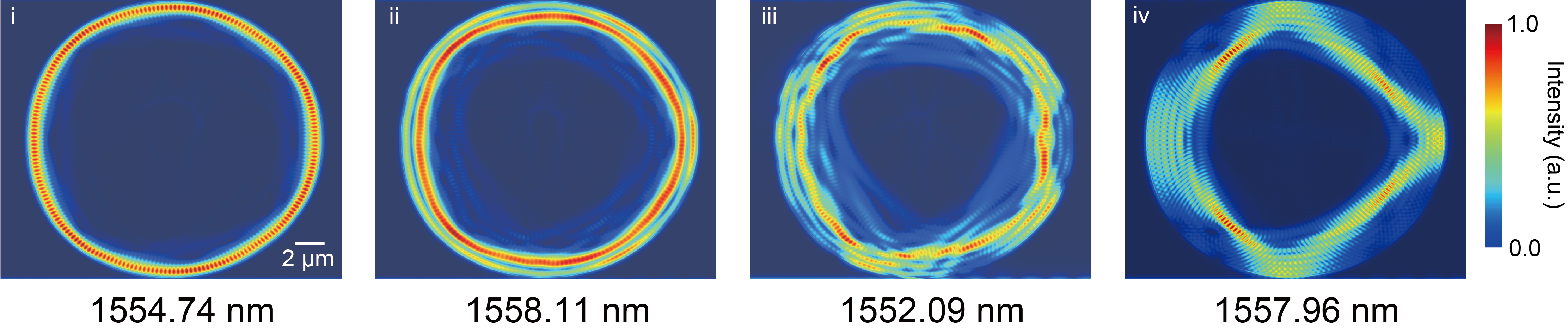


**Fig. S7 |** Numerically calculated electric-field distributions of certain periodic/quasi-periodic resonant modes, corresponding to Husimi maps in manuscript Fig. **2d**.

**S6. Chip photo**

As illustrated in Fig. **S8** our chaos-assisted spectrometer and reference waveguide are labeled on chip optical microscopy photo by black solid square and yellow dashed lines, respectively. The measured transmission spectrum of the reference waveguide is illustrated in Fig. **S9**. This insertion loss includes coupling losses of two grating couplers, energy outgoing from CW laser, measurement system loss such as polarization controller, fibers, and 3-dB couplers, as well as propagation loss, etc.


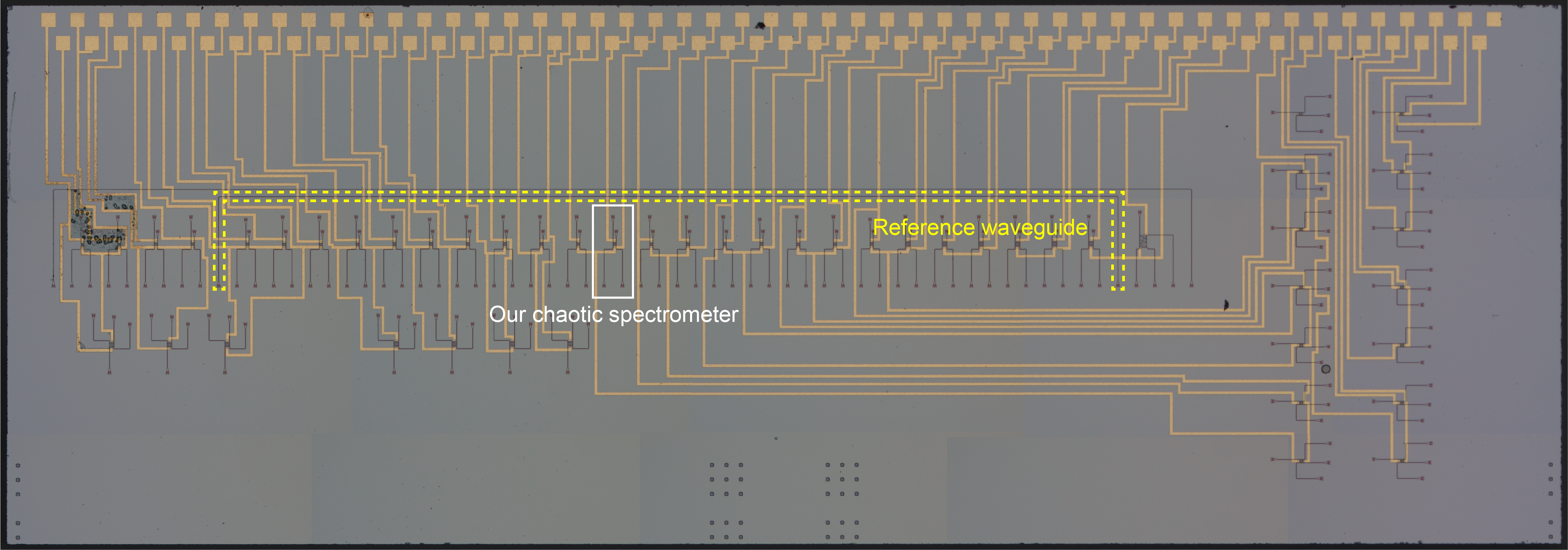


**Fig. S8 |** Detailed optical microscopy image of the fabricated on-chip chaos-assisted spectrometer devices.


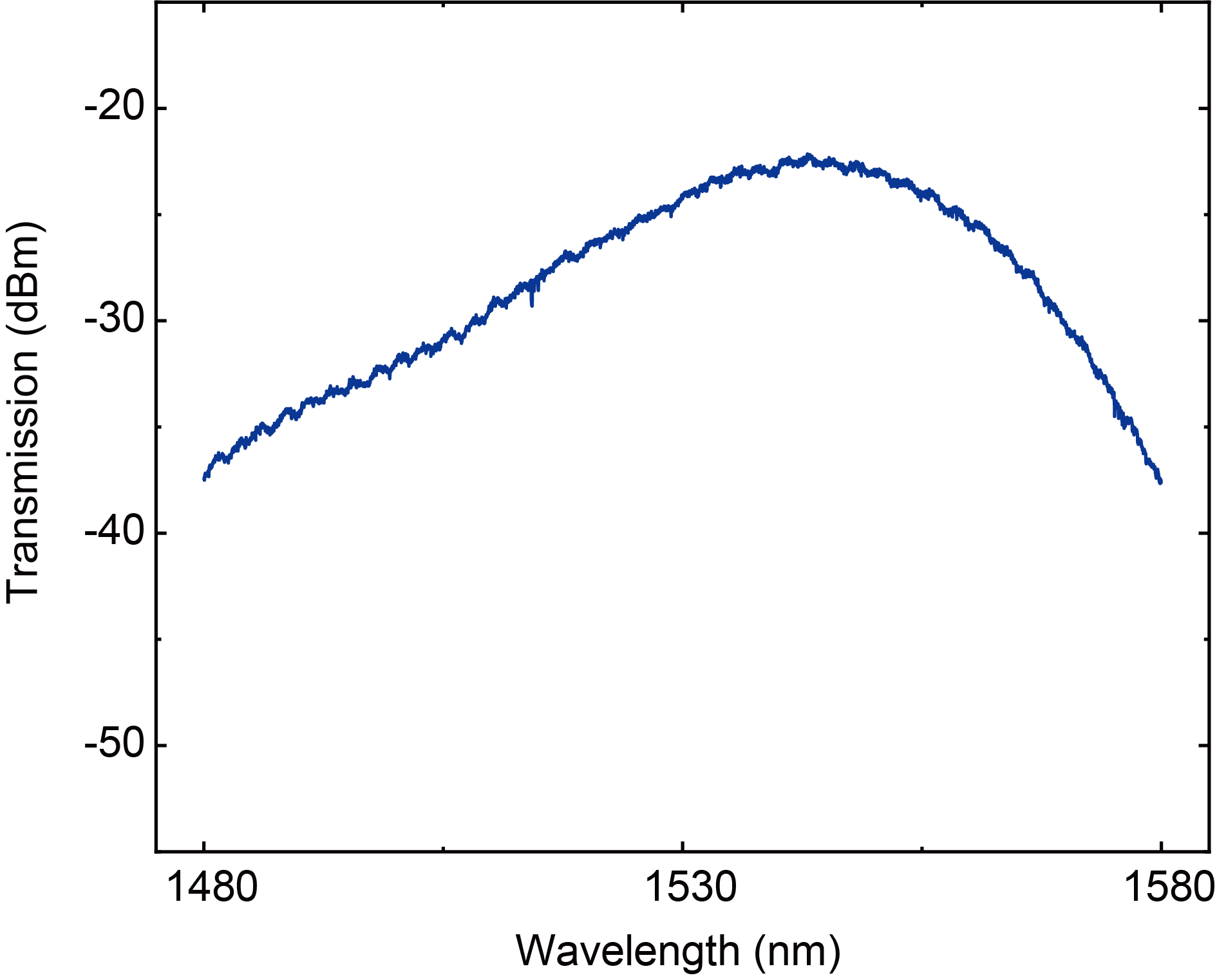


**Fig. S9 |** Measured transmission spectrum of the straight waveguide.

**S7.** **High Q-factor of narrow resonance peaks in transmission spectra**

Compared to microring resonators, the chaotic cavity which is a smoothly deformed microdisk resonator facilitates the propagation of resonant modes entirely confined by the boundary because of total internal reflection. Hence, a higher intrinsic *Q*_int_ is promised, attributed to the noteworthy reduction in Rayleigh scattering loss arising from a purely rough sidewall during fabrication. Under our moderate deformation condition, a considerable number of rays remain constrained within invariant curves. Consequently, phase-space diffusion tends to proceed slowly, resulting in only a minor reduction of the Q-factor for the corresponding resonant modes (Q spoiling)^1^. Therefore, high Q-factor features are still promised. The Q-factors of resonance peaks marked by black arrows in Fig. **3d** are illustrated in Table. **S1**.

**Table. S1.** Q-factors of resonance peaks marked by black arrows in Fig. **3d**.

| Number | 1 | 2 | 3 | 4 | 5 | 6 | 7 | 8 |
| --- | --- | --- | --- | --- | --- | --- | --- | --- |
| Wavelength (nm) | **1483.48** | **1493.27** | **1498.75** | **1500.08** | **1508.35** | **1518.08** | **1525.83** | **1527.94** |
| Bandwidth (pm) | 30.6 | 26.8 | 24.5 | 22.9 | 24.9 | 25.1 | 32.0 | 30.7 |
| Q-factor | 50637 | 57812 | 63357 | 67704 | 62374 | 61765 | 48426 | 50499 |

| Number | 9 | 10 | 11 | 12 | 13 | 14 | 15 |
| --- | --- | --- | --- | --- | --- | --- | --- |
| Wavelength (nm) | **1533.85** | **1536.34** | **1537.94** | **1550.50** | **1558.40** | **1561.04** | **1571.74** |
| Bandwidth (pm) | 31.3 | 30.5 | 30.9 | 31.9 | 34.3 | 26.4 | 25.3 |
| Q-factor | 49598 | 50842 | 50150 | 48638 | 45162 | 58611 | 61312 |

**S8. Temporally decorrelation**

For our chaos-assisted spectrometer, the chaotic cavity can contain more diverse resonant modes. These resonant modes possess the various FSRs, promising the sampling decorrelation of our chaos-assisted spectrometer. Here, we randomly select two wavelength channels at 1499.5 nm and 1560.3 nm to present the sampling decorrelation, as exhibited in Fig. **S10**. The low overlapping of the adjacent wavelength channels can ensure sufficient decorrelation and the number of sampling channels must be reduced to permit the low overlapping. The standard is the position disparity of two peaks in the adjacent wavelength channels needs to exceed the full width at half maximum (FWHM) of two peaks. In Fig. **S10**, we provide two selected wavelength channels and the corresponding adjacent wavelength channels at λ_1_ of 1499.5 nm and λ_2_ of 1560.3 nm, marked as blue and red lines where interval δ*λ* of the adjacent wavelength channels is the estimated resolution (40 pm) from the auto-correlation function of our chaotic cavity, evidencing the low overlapping of the adjacent wavelength channels in our chaos-assisted spectrometer.


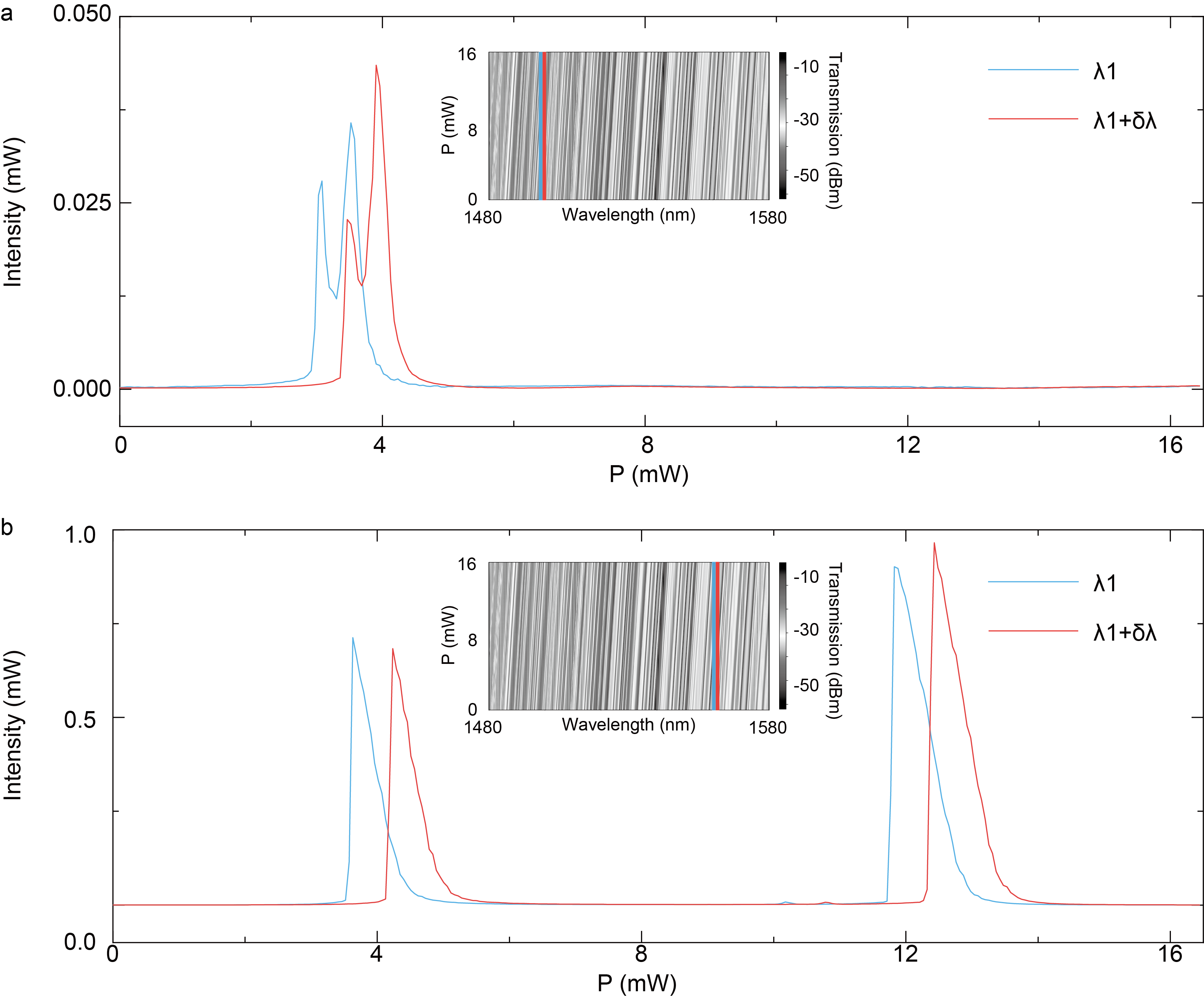


**Fig. S10 |** Measured scanning traces at two selected wavelength channels and the corresponding adjacent wavelength channels at λ_1_ of 1520 nm (**a**) and λ_2_ of 1570 nm (**b**), the interval of adjacent wavelength channels marked as blue and red lines is δλ.

**S9. Estimated resolution comparison of the chaotic cavity and microdisk resonator**

Here, we present the correlation function and estimated resolution in Fig. **S11** for the chaotic cavity and circular microdisk, both of which have the same size and coupling gaps in the coupling region. The FWHM of the auto-correlation function can represent the possible resolution of the spectrometer because the more rapidly fluctuating response matrix could be capable of distinguishing more adjacent wavelength peaks. It’s noted that the FWHM reflects the overall spectral sharpness and the decorrelation level between wavelength channels, which highly corresponds to the Q-factor. They exhibit similar estimated spectral resolution values due to their comparable Q-factors. In our design, the coupling gap is set to a moderate value that the *Q*_load_ is predominantly influenced by *Q*_ext_ ($Q_{\mathrm{load}}^{-1}=Q_{\mathrm{int}}^{-1}+Q_{\mathrm{ext}}^{-1}$), where *Q*_ext_ reflects the degree of external power loss due to coupling, signifying that the Q-factor is primarily determined by the coupling gaps in the resonators. Therefore, the spectral resolution is dominated by the coupling gap, while the deformation degree (with *α* ranging from 0 to 0.4) has a negligible impact on it. When maintaining the same configuration, the spectrometer devices with a similar Q-factor generally possess a similar resolution whether the device is fabricated by a chaotic cavity or a circular microdisk.


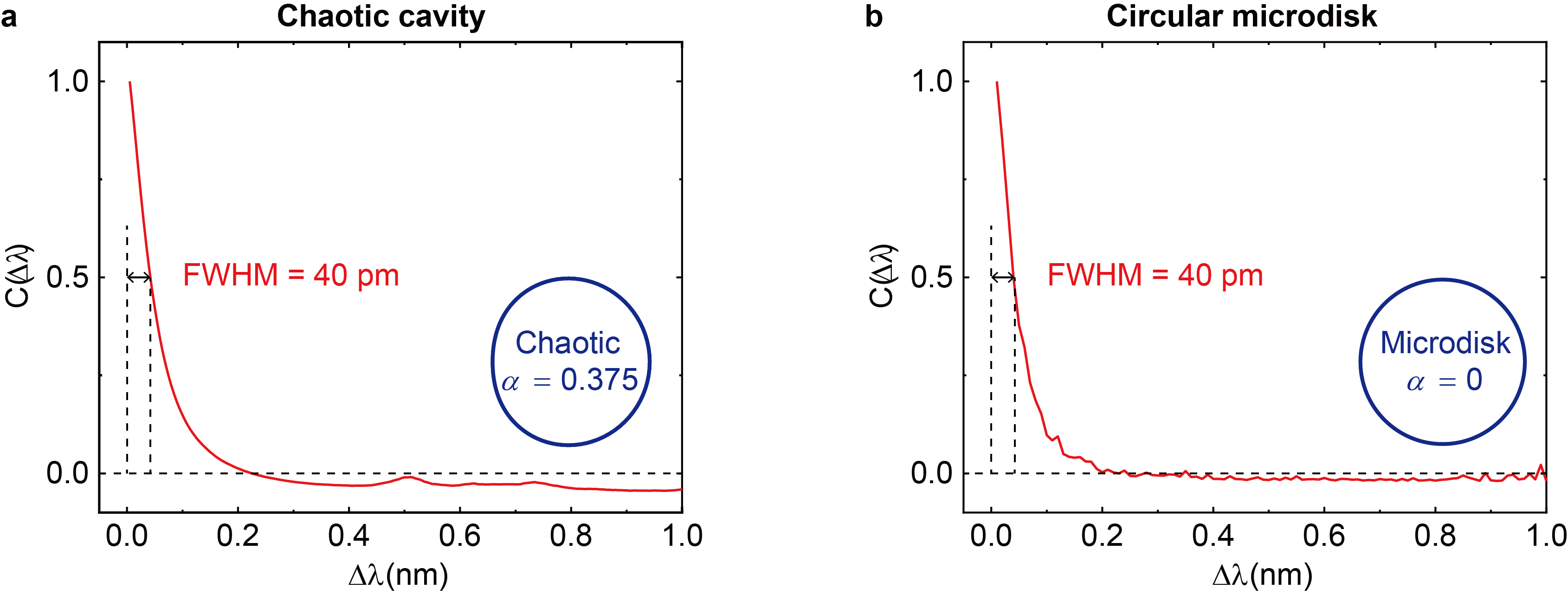


**Fig. S11 |** Spectral correlation function of C(Δλ) around Δλ=0 for **a,** chaotic cavity and **b,** circular microdisk. They yield the same size and coupling gap. The estimated resolution is illustrated by the black dashed line.

The essential difference between our chaotic cavity and a circular microdisk is the holistic performance on spectra analysis due to the emphatic destruction of the comprehensive quality of the response matrix. As detailed in Supplementary Information **S4** and **S13**, our chaos-assisted spectrometer exhibits a lower periodicity level of auto-correlation function and lower condition number compared to other spectrometers based on a circular microdisk or microring. Hence, our chaos-assisted spectrometer demonstrates the more powerful spectra recognition capability, the lower reconstruction error, and the stronger tolerance of system noise. Despite the Q-factor of the device corresponding to the minimum resolution level, it’s not the only factor determining resolution in the reconstruction process. Various published literatures^2–5^ have demonstrated the actual resolution of the spectrometer can also be jointly influenced by the spectral pixels, the number of sampling channels, algorithm performance, OSNR, etc.

**S10. Numerical analysis**

We now conduct a numerical analysis of the experimentally pre-calibrated response matrix **T**. When tackling measurement noise in a real-world ill-conditioned inverse problem, it is imperative to incorporate prior knowledge or assumptions as regularization terms. To assess the quality of our response matrix and the extent to which decoding requires prior knowledge, we employ singular-value decomposition (SVD) on matrix **T**, where SVD can be executed to uniquely decompose the response matrix **T** into a diagonal matrix (**Σ**) and two complex unitary matrices (**U** and **V**):

$$\begin{aligned} \mathbf{T}_{N_{p}\times M_{w}}=\mathbf{U}_{N_{p}\times N_{p}}\boldsymbol{\Sigma}_{N_{p}\times M_{w}}\mathbf{V}_{M_{w}\times M_{w}}^{*}\#\left( 1 \right) \end{aligned}$$

where **Σ** = *diag*[*σ*_1_*, ..., σ_n_*], **U** and **V** are respectively composed of left- and right-singular vectors in the response matrix **T**. Hence, $\mathbf{I}^{\boldsymbol{\dagger}}$**,** the pseudo-inverse of **I**, can be described as:

$\begin{aligned} \mathbf{I}^{\boldsymbol{\dagger}}=\sum_{i=1}^{N_{p}} \frac{\boldsymbol{u}_{i}^{*}\boldsymbol{\Sigma}}{\sigma_{i}}\boldsymbol{v}_{i}\boldsymbol{\#}\left( 8 \right) \end{aligned}$where *σ_i_*, arranged in descending order, refers to the singular value of **T**, ***u****_i_* and ***v****_i_* are *i*th singular vectors of **U** and **V**. The decay rate of the singular values *σ_i_* is employed to evaluate the quality of the response matrix, wherein the faster decay necessitates greater reliance on priori information to counteract ill-posed conditions. The normalized singular value *σ_i_ / σ*_max_ of our spectrometer is represented in black dots, in comparison with other studies as green and purple dots are presented in Fig. **S12a**. Our work realizes smooth and flat singular values with a remarkable reduction in decay, highlighting the robustness of the response matrix to noise. The condition number, which can reveal the orthogonality of the response matrix, is computed as $\kappa\left( \mathbf{T} \right)=\left\| \mathbf{T} \right\|_{2}\left\| \mathbf{T}^{-1} \right\|_{2}={\sigma_{\max}}/{\sigma_{\min}}$. Condition number serves as a pivotal performance indicator that directly evaluates the inherent property of a matrix, providing a bound on the inaccuracy of the solution after approximation. Considering the measurement noise *δ***S**, the level of $\mathbf{I}^{\boldsymbol{\dagger}}$influenced by noise is expressed as:

$$\begin{aligned} \frac{\left\| \mathbf{I}^{\dagger}-\mathbf{I} \right\|}{\left\| \mathbf{I} \right\|}\leq\kappa\left( \mathbf{T} \right)\frac{\left\| \delta\mathbf{S} \right\|}{\left\| \mathbf{S} \right\|}\#\left( 9 \right) \end{aligned}$$

Consequently, a large condition number signifies that even slight perturbations in the detected power **S** can significantly degrade the reconstruction accuracy, which is called an ill-conditioned problem. Consequently, even advanced algorithms encounter greater challenges in the inversion of such numerically unstable matrices. Besides, the condition number reflects how quickly an iterative method, such as the conjugate gradient iteration, will converge. Furthermore, the condition number directly reveals the degree of regularization required, reflecting the extent of reliance on software and algorithmic assistance, as well as the incorporation of prior assumptions^6^. Our condition number of the chaos-assisted spectrometer is only 40.16, which is several orders of magnitude smaller than the other two published works of reconstruction spectrometers as listed below.

| **Resource** | $\kappa\left( \mathbf{T} \right)$ |
| --- | --- |
| Yang^7^ | 1.474×10^4^ |
| Yoon^8^ | 3.631×10^5^ |
| Our work | 40.16 |

These numerical analyses demonstrate the quasi-orthogonality of the response matrix, demonstrating its superior robustness to measurement noise and the stable performance of our chaos-assisted spectrometer. Fig. **S12b** presents the Fast Fourier Transform (FFT) applied to the left singular vector (FFT(**U**)), enabling the potential collection of comprehensive information with fewer channels due to FFT(**U**) covers a broad distribution from low to high frequency.


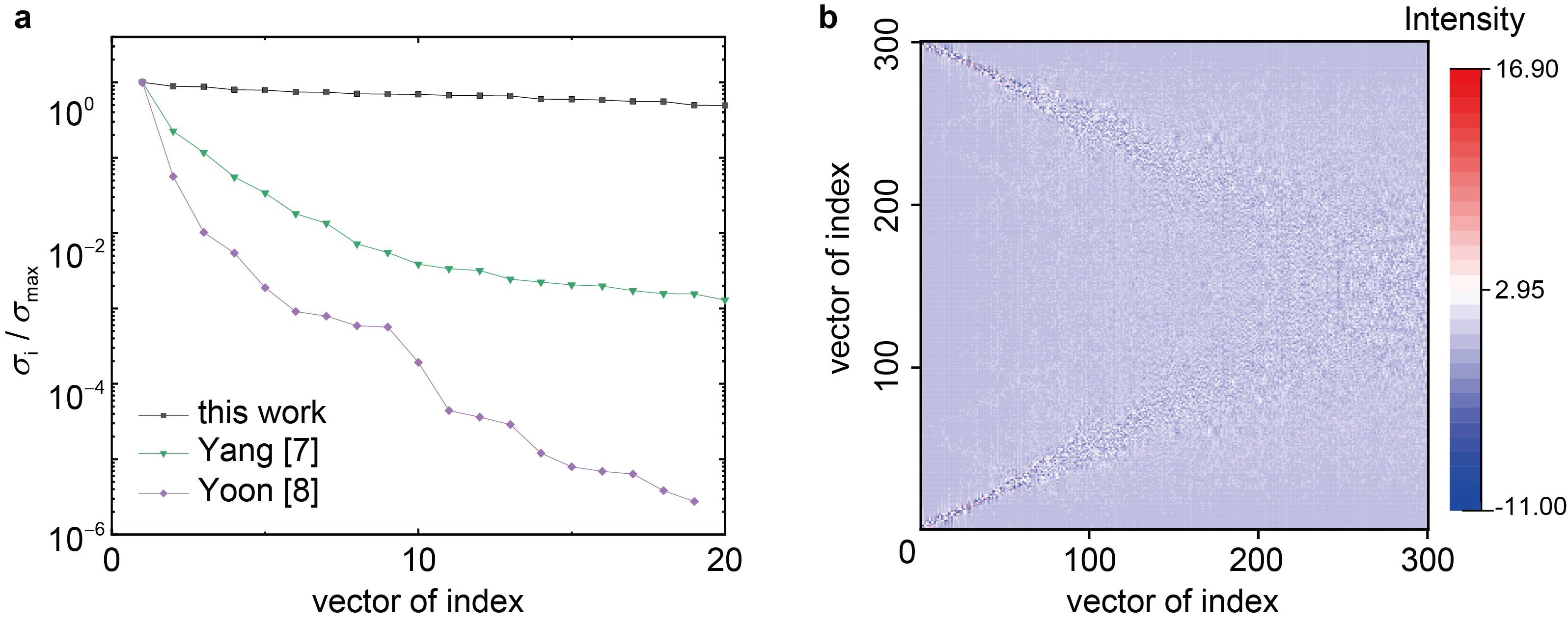


**Fig. S12 |** **a,** Calculated singular value *σ_i_*/*σ*_max_ of experimentally pre-calibrated response matrix **T** of the chaos-assisted spectrometer (black lines) and other published works (green and purple lines, respectively). **b**, Calculated FFT of singular vector in left unitary matrix **U** of **T**.

Moreover, *Picard plots* with *σ_i_*, ***u****_i_^*^***I,** and ***u****_i_*^*^**I**/*σ_i_* are calculated to confirm the convergence of solutions to the linear inverse problem, as shown in Fig. **S13**, wherein the probe signals are Gaussian, square, and spike signals. The SVD coefficients ***u****_i_^*^***I** exhibit a more rapid decay compared to the singular values initially, plateauing for *i* ≥ 100 due to the determination of noise. It can also be supported by observing the initial decay of the solution coefficients ***u****_i_^*^***I**/*σ_i_* and the absence of a noticeable increase even at a higher vector of index. For the larger vector index, it is advisable to disregard the part of the *Picard plot* corresponding to tiny singular values where ***u****_i_^*^***I** level off at some noise plateau^9^. Therefore, for our chaos-assisted spectrometer, the discrete Picard condition is fulfilled for various kinds of archetype signals, and a bounded and convergent solution with a practical norm definitely exists due to the faster average decays of ***u****_i_*^*^**I** than *σ_i_*.


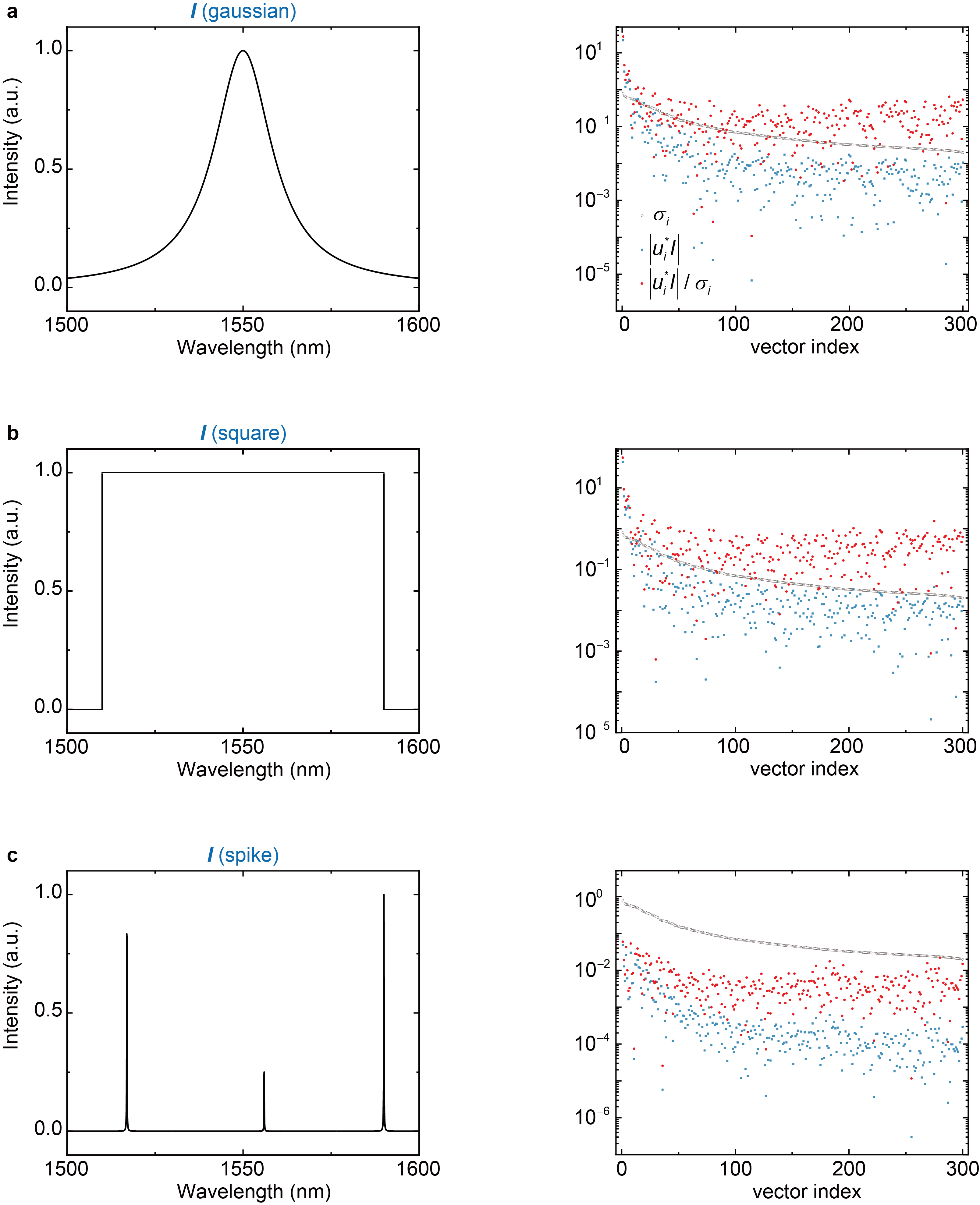


**Fig. S13 |** *Picard plots*. Spectra of test signals (left column) and calculated *Picard plots* (right column) of **a,** gaussian signal; **b**, square signal; and **c**, spike signal.

**S11. Numerical simulation based on measurement response matrix**

Numerically calculated results are conducted to evaluate the effectiveness of the suggested spectrometer in extracting a sequence of probe signals from three representative spectra: discrete (Fig. **S14a**), continuous (Fig. **S14b**), and step signal (Fig. **S14c**). We mathematically build probe signals of *M*_w_ wavelength points whose distributions are shown in the first column of Fig. **S14** with blue backgrounds to be inputted into the measured response matrix. The numerically simulated optical power in varying heating power channels can be calculated by $\mathbf{S}_{N_{p}\times1}=\mathbf{T}_{N_{p}\times M_{w}}\mathbf{I}_{M_{w}\times1}$. We first directly solve the under-determined linear algebra function of equation (10) to reconstruct the probe signals. The reconstructed spectrum $\mathbf{I}^{\dagger}$ and the corresponding relative errors *e* are calculated and illustrated in the middle row of Fig. **S14** with yellow backgrounds.

It is critically noted that the response matrix, which has been calibrated through experimentation, is ill-posed with *N* < *M* so that the pre-existing information is required for regularization to counterbalance the issue of ill-conditioning. Even without regularization, discrete signals can be resolved with great accuracy of minimal error. Yet, continuous and step probe signals are prone to significant misinterpretation, causing considerable relative inaccuracy as shown in the center figures of Fig. **S14b** and Fig. **S14c**. Appropriate regularization is crucial when solving continuous signals even without measurement noise, while this requirement can be bypassed for discrete signals because of the sparsity principle. For discrete signals and continuous signal processing, we respectively employ $\alpha_{1}\left\| \mathbf{I} \right\|_{1}$ and Tikhonov regularization of $\alpha_{3}\left\| D_{2}\mathbf{I} \right\|_{2}$ as the regularization term. For the step signal processing, total variation (TV) regularization and Tikhonov regularization are introduced simultaneously of $\alpha_{2}\left\| D_{1}\mathbf{I} \right\|_{1}+\alpha_{3}\left\| D_{2}\mathbf{I} \right\|_{2}$. TV smoothing supports the presence of steep gradients, whereas the *l*_2_-norm of the first derivative of **I** does not permit steep gradients, resulting in the existence of a highly smooth solution. Therefore, the piecewise smoothing solution is produced by TV regularization. The appropriately regularized reconstruction results are depicted in the third column of Fig. **S14**, achieving the significant inhibition of relative errors. It clearly indicates that the empirically calculated response matrix is capable of effectively processing various archetypal signals with the assistance of suitable regularization techniques. Therefore, under a sufficiently stable experimental environment, our suggested encoder can accurately differentiate and recover complex incident spectra with a diversity of optical characteristics.


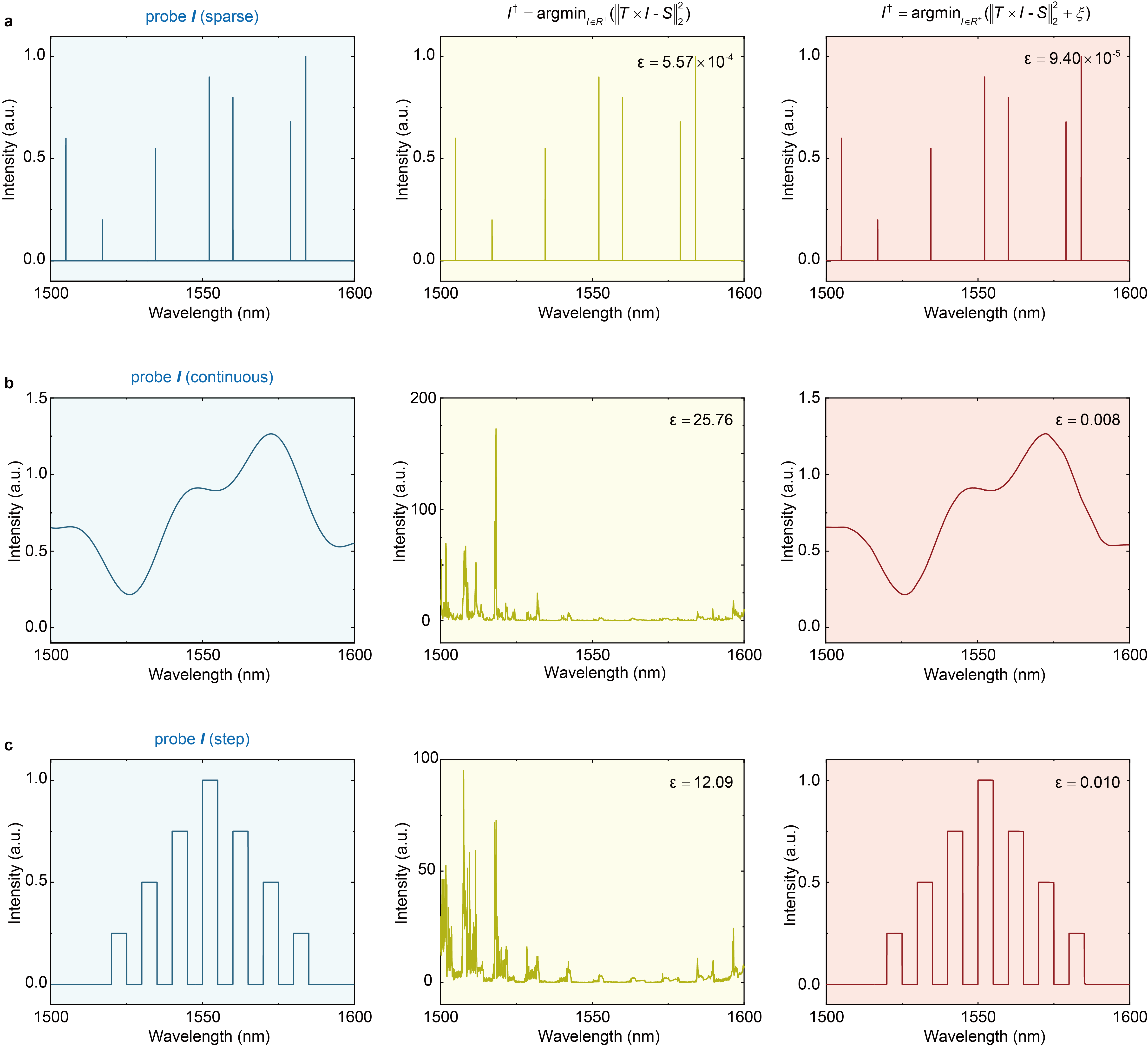


**Fig. S14 |** Numerical reconstruction of test spectra with **a**, discrete; **b**, continuous; and **c**, step features. First column with blue backgrounds: test input spectra. Second and third columns with yellow and red backgrounds respectively: Reconstruction results with and without regularization coefficients, where *ξ* refers to appropriate regularization.

**S12. Noise tolerance**

We numerically test the robustness of the response matrix against noise by adding white noise that follows Gaussian distribution to the measurement. To quantify the noise level, we adopt the same indicator as Zongyin Yang et al^7^., specifically the coefficient of variation (CV), which is the ratio of the standard deviation (*σ*) to the mean (*µ*). The reconstruction results for a sparse discrete signal with varying peak intensities are illustrated in Fig. **S15** under different noise levels ranging from 2% to 40%. Notably, at a noise level of 25%, the discrete signals can still be recognized with an acceptable error margin, which reveals strong robustness of our spectrometer.


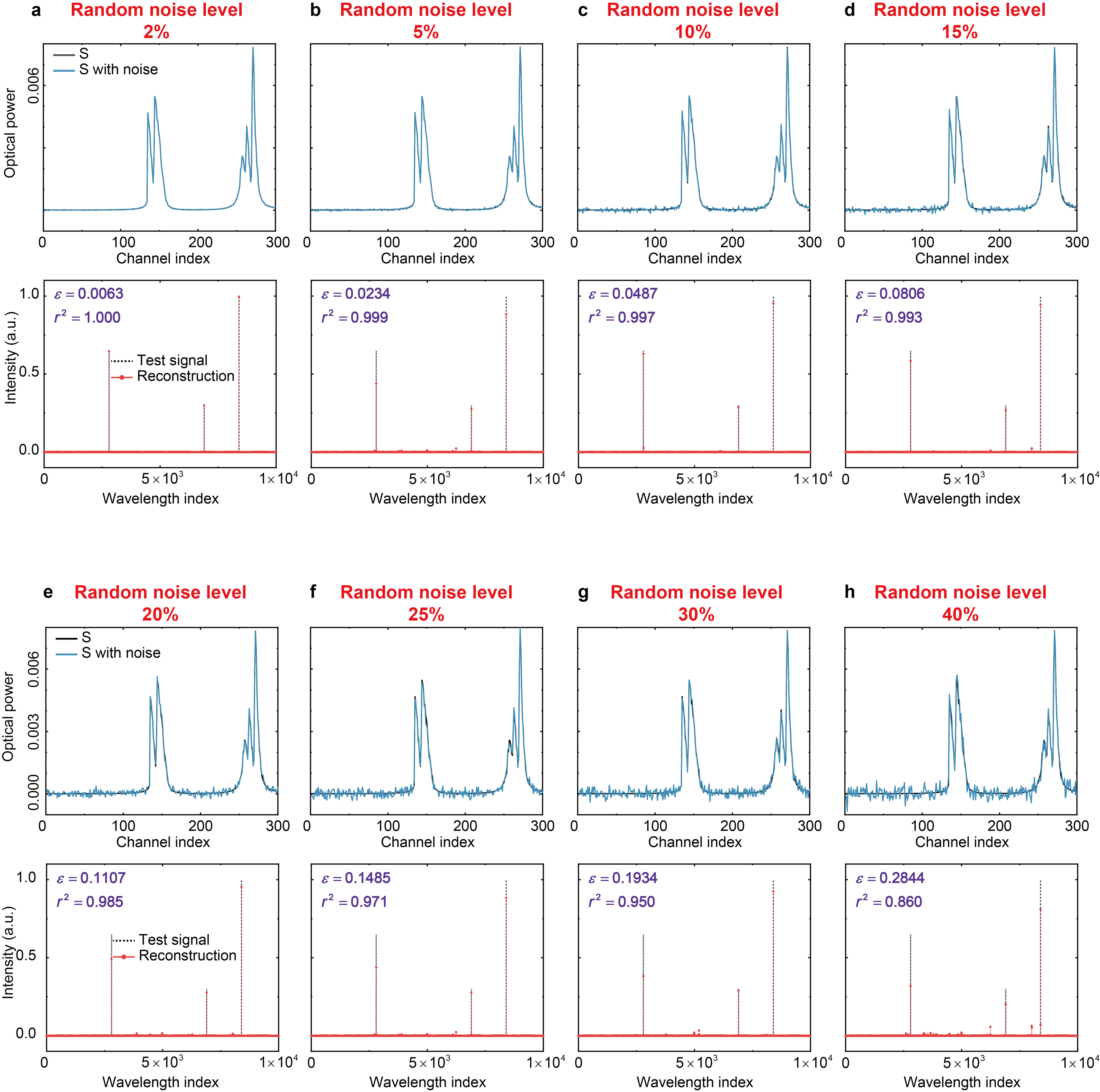


**Fig. S15 |** Simulated reconstruction results for a series of discrete signals under different levels of measurement noise. **a** to **h**, noise level ranging from 2% to 40%. Top row: detected optical power with and without noise; bottom row: Test signal and reconstructed signal.

If we adopt only 1000 spectral channels, sacrificing the spectral resolution to 100 pm, the reconstruction results illustrated in Fig. **S16** demonstrate that the reduced response matrix exhibits remarkable robustness against measurement noise, withstanding levels up to 60%. This further highlights the high quality of the response matrix. On the other hand, this reveals the sacrifice we made involved in choosing high spectral resolution and high compression ratio demonstrated in the manuscript.


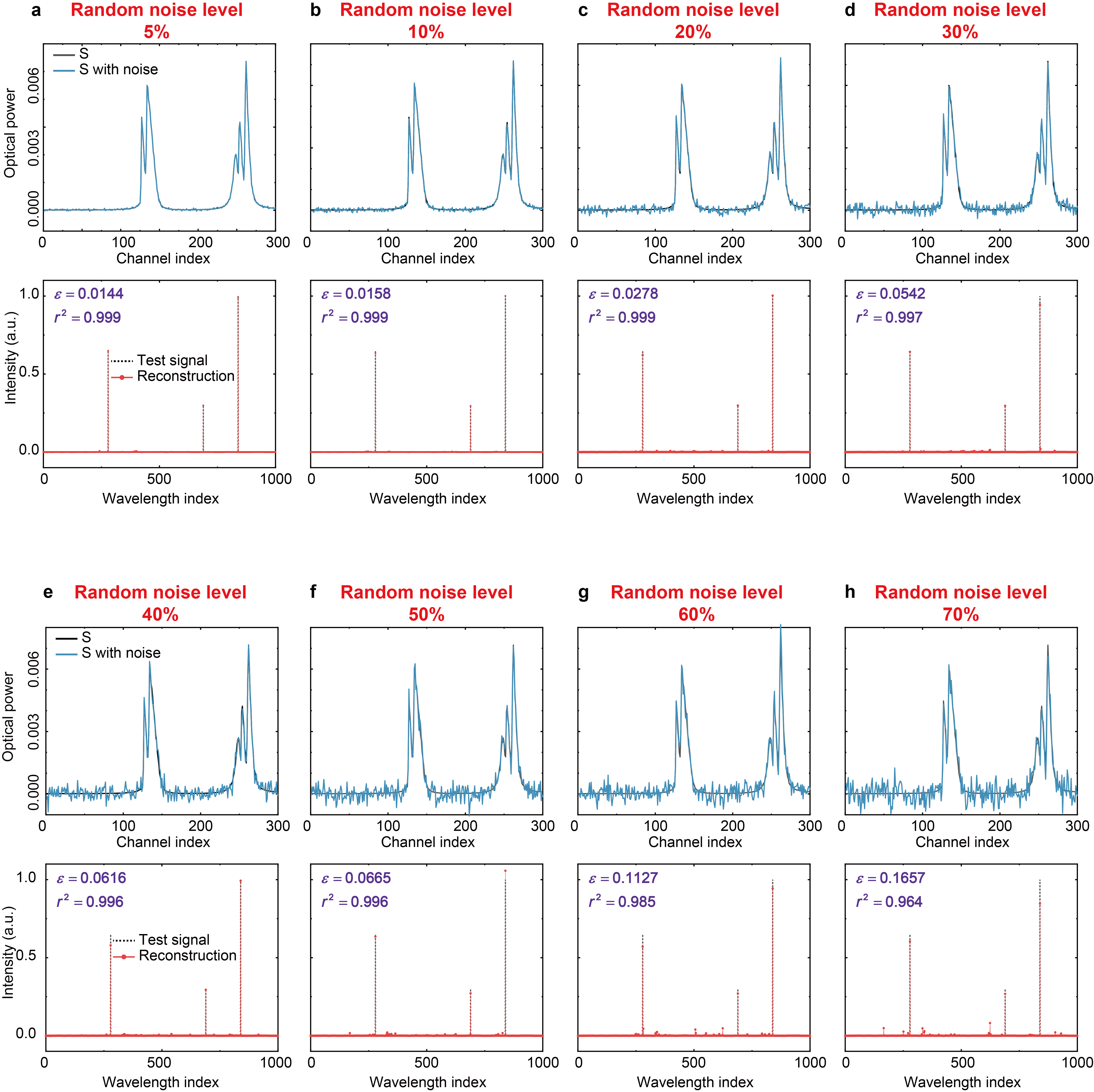


**Fig. S16 |** Simulated reconstruction results for a series of discrete signals under different levels of measurement noise. The spectral channel number is 1000, with 100 pm wavelength grid. **a** to **h**, noise level ranging from 5% to 70%. Top row: detected optical power with and without noise; bottom row: Test signal and reconstructed signal.

We extend noise tolerance analysis to continuous signals, providing a summary of the relative errors for both continuous and multi-peak discrete signals under the same levels of white noise superimposed on the measurement **S**, as depicted in Fig. **S17.** Despite the higher number of sampling channels for continuous signals, continuous signals demonstrate lower reconstruction accuracy and more fragile noise tolerance. This numerical analysis further elucidates the reasons underlying the comparatively inferior reconstruction of continuous signals versus discrete signals.


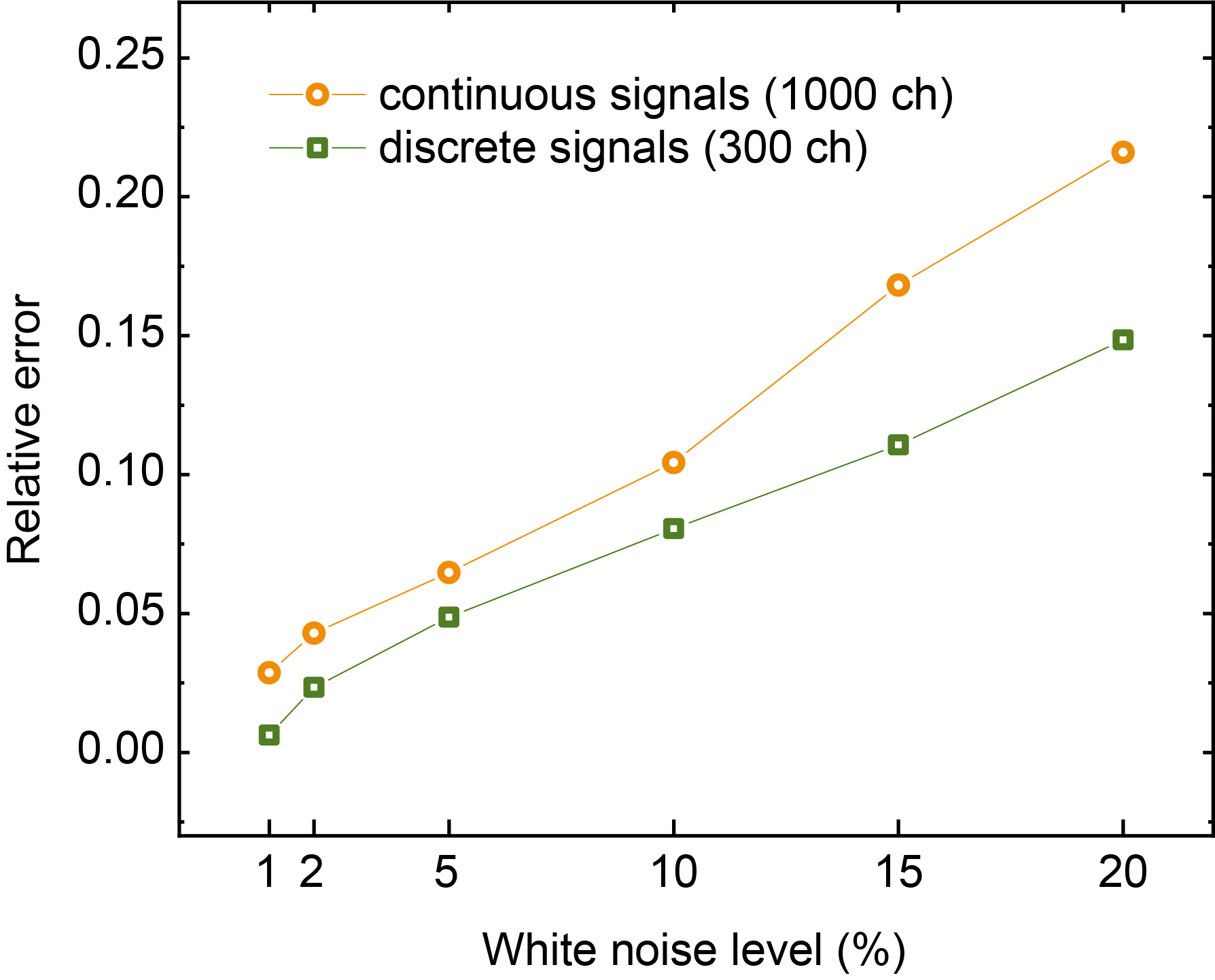


**Fig. S17 |** Relative errors (ε) of multi-peak discrete signal (green points) and continuous signal (orange points) under different levels of white noise are added in to the measurement (**S**), respectively.

**S13. Performance comparison of the optimal chaos-assisted spectrometer and other micro-cavity structures**

A performance comparison analysis is conducted between the chaos-assisted spectrometer (*α* = 0.375) and other systems, including the microring resonator, microdisk resonator, and other chaotic cavities with *α* = 0.3 and 0.45, validating the optimal deformation parameter and demonstrating the superior of our chaos-assisted spectrometer compared to conventional resonators (microring/microdisk resonators).

We utilize the measured transmission spectrum from the drop port under zero driving power to numerically construct a response matrix with 300 heating channels, matching the configuration of our experimentally obtained matrix. By thermo-optical tuning through 3D-FDTD, we ensure that the spectral response under zero driving power could undergo a wavelength red-shift degree equivalent to our experimentally obtained matrix for each heating power and each wavelength point. These response matrices would be almost consistent with experiments, as we only numerically simulated the wavelength red-shift process and they are predominant by experimentally measured transmission spectrum under zero driving power. For the response matrix of the chaotic cavity of *α* = 0.375, we directly utilize the experimental data. For the response matrix of microring resonators, we utilize simulation results from 3D-FDTD. Here, based on these matrices, we provide the simulation results of spectra reconstruction for microring resonator, microdisk resonator, and chaotic cavities with different α of 0.3, 0.375, and 0.45.

The response matrices of microring resonator, microdisk resonator, and chaotic cavities with different *α* of 0.3, 0.375, and 0.45 are exhibited in Fig. **S18a**, spanning from the first row to the 5th row in order. These matrices maintain the comparable Q-factors with maximum values of 78828, 77159, 51667, 67704, and 72864, and average values of 49831, 33246, 26152, 25471, and 25636, respectively. Condition numbers of these response matrices are summarized in Table. **S2**.

**Table. S2**. Calculated condition numbers of response matrices for the different structures.

| **Scheme** | **Condition number** |
| --- | --- |
| Microring | 223.41 |
| Microdisk | 188.17 |
| Chaotic cavity (*α* = 0.3) | 72.71 |
| Our chaotic cavity (*α* = 0.375) | 40.16 |
| Chaotic cavity (*α* = 0.45) | 496.61 |

Here, we construct a discrete signal with adjacent multi-peak signals in the wavelength domain for the resolvability comparison; and a sparse discrete signal with peaks of different intensities across the whole operational bandwidth for the validation of matrix completeness and overall performance verification. Different levels of white noise are loaded into the measurements (**S**) for better identification of the reconstruction performance of these structures.

The resolvability comparison is illustrated in Fig. **S18** with different noise levels of 0% (**b**), 2% (**c**), and 5% (**d**), where the reconstruction results of microring resonator, microdisk resonator, and chaotic cavities with different *α* of 0.3, 0.375, and 0.45 are marked on different colored backgrounds of blue, cyan, green, red, and yellow, respectively. Due to the excessive periodicity, the microring can’t even correctly recognize the incident signals without noise (blue background). The microdisk possesses a certain resolvability for these signals without noise but quickly loses the recognition ability with the loading of noise (cyan background). The chaotic cavity with parameter *α* of 0.3 can address the resolvability for these signals (green background), however, all the corresponding reconstruction errors are higher than the chaotic cavity with parameter *α* of 0.375 for each loading noise level. The chaotic cavity with parameter *α* of 0.45 has completely no recognition ability for the adjacent multi-peak discrete signals even without loading noise (yellow background). The chaotic cavity could still retain the powerful spectra resolvability even with noise (red background). When loading the adjacent multi-peak discrete signals mixed with the noise of 5% level, the reconstruction error of the chaotic cavity is merely 0.0776, as exhibited in Fig. **S18d**, red background.


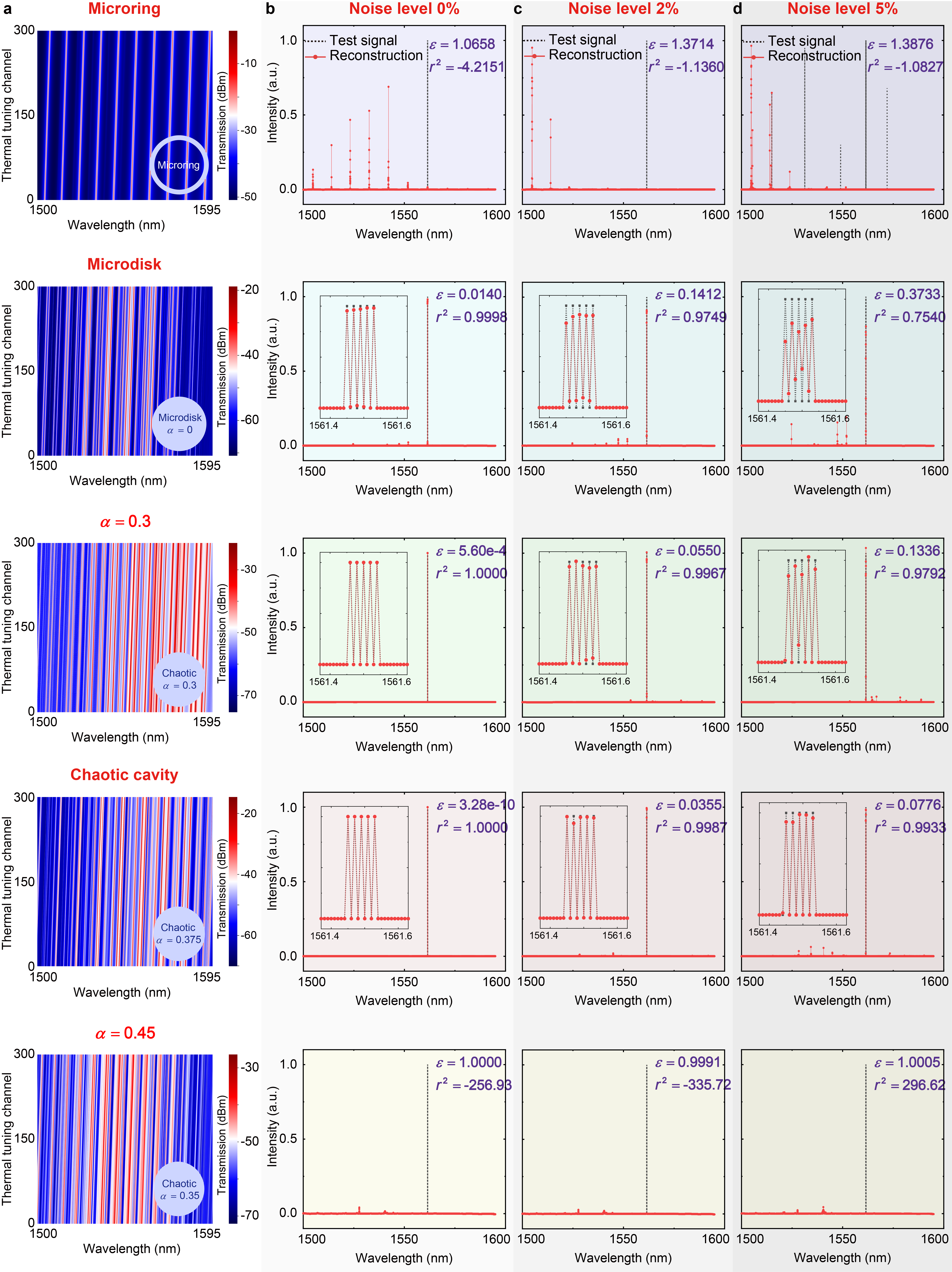


**Fig. S18 |** **a,** Response matrices of microring resonator (1^st^ row), microdisk resonator (2^nd^ row), chaotic cavities with different deformation parameters *α* of 0.3 (3^rd^ row), 0.375 (4^th^ row), and 0.45 (5^th^ row). Resolvability comparison for a discrete signal with adjacent multi-peak signals in the wavelength domain. Measurements (**S**) are mixed with noise of 0% level (**b**), 2% level (**c**), and 5% level (**d**). Reconstruction results of microring resonator, microdisk resonator, and chaotic cavities with different *α* of 0.3, 0.375, and 0.45 are marked on different colored backgrounds of blue (1^st^ row), cyan (2^nd^ row), green (3^rd^ row), red (4^th^ row), and yellow (5^th^ row), respectively.

The reconstruction performance comparison of the sparse discrete signals is illustrated in Fig. **S19**, with different noise levels of 0% (**a**), 1% (**b**), and 3% (**c**). The performance of microring is worst to be disabled for the reconstruction of the sparse discrete signal without mixing noise due to the huge periodicity level of response sampling, as shown in the first row of Fig. **S19a**. Despite the microdisk with a few resonant modes can possess a low reconstruction error for the sparse discrete signal without mixing noise, the reconstruction error would rapidly increase with the noise level rising. The chaotic cavity with parameter *α* of 0.3 can address this test signal (green backgrounds), however, exhibiting an apparent lower tolerance for measurement noises compared to our optimal chaotic cavity device (*α* = 0.375, red backgrounds). The chaotic cavity with *α* = 0.45 exposes a weak recognition ability that the majority of the signal peaks cannot be interpreted (yellow backgrounds). The chaotic cavity can maintain the reconstruction accuracy for these noise levels, where the reconstruction error is still only 0.1014 when mixing the noise of 3% noise level, as represented in Fig. **S19c**, red background.


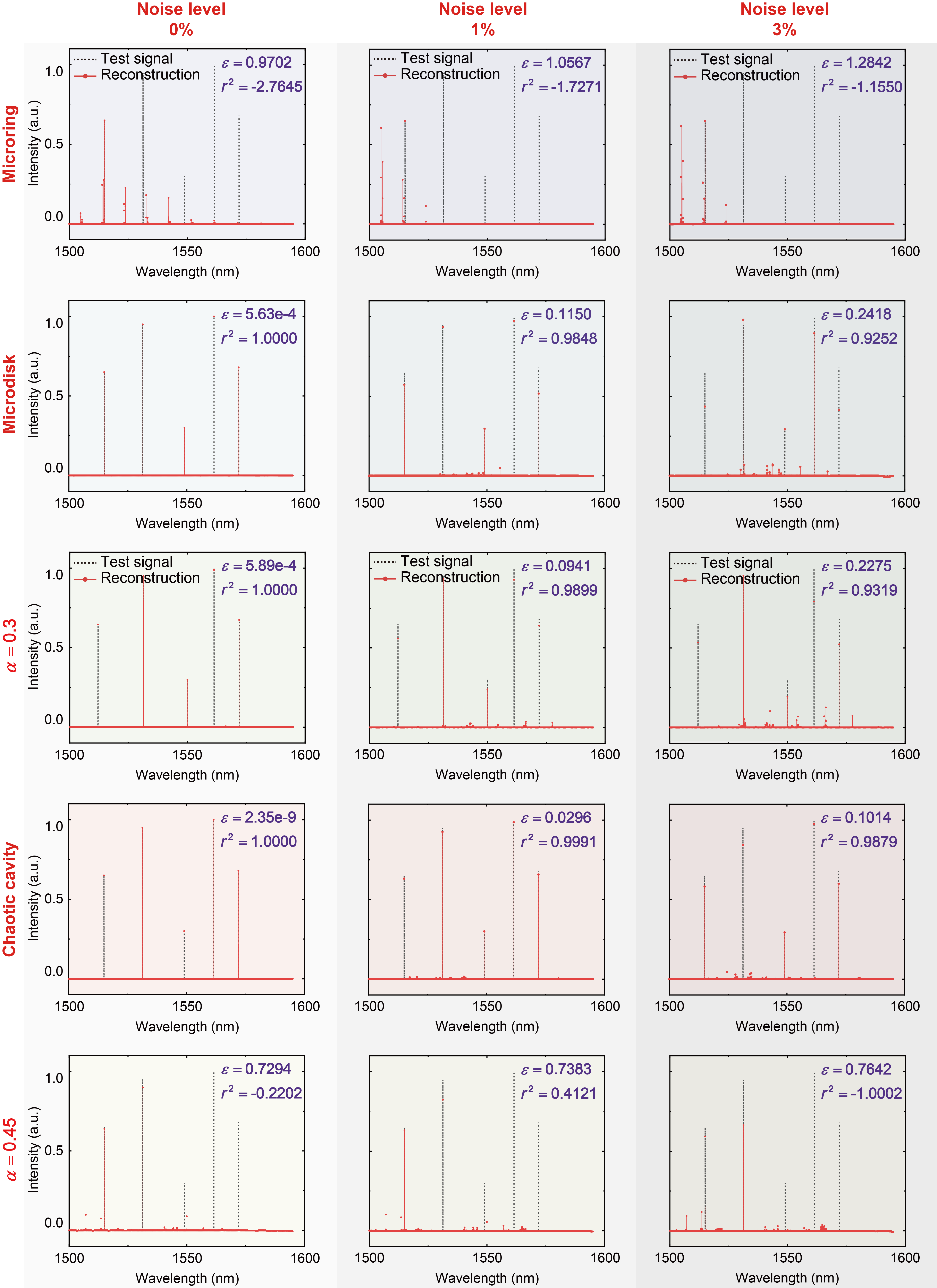


**Fig. S19 |** Reconstruction performance comparison for a sparse discrete signal with varying peak intensities across the whole bandwidth. Measurements (**S**) are mixed with noise of 0% level (**a**), 1% level (**b**), and 3% level (**c**). Reconstruction results of microring resonator, microdisk resonator, and chaotic cavities with different *α* of 0.3, 0.375, and 0.45 are marked on different colored backgrounds of blue (1^st^ row), cyan (2^nd^ row), green (3^rd^ row), red (4^th^ row), and yellow (5^th^ row), respectively.

We summarize the condition numbers of response matrices for microring, microdisk, and chaotic cavities with *α* = 0.3, 0.375 (optimal chaotic cavity), and 0.45 in Fig. **S20a**. The relative errors of reconstruction results of two types of representative signals for these five cavities under different noise levels are summarized in Fig. **S20b** and Fig. **S20c**, respectively. Apparently, the chaotic cavity with *α* = 0.375 delivers the superior reconstruction capability and strongest robustness against measurement noise compared to other conventional cavities and chaotic cavities with different α values.


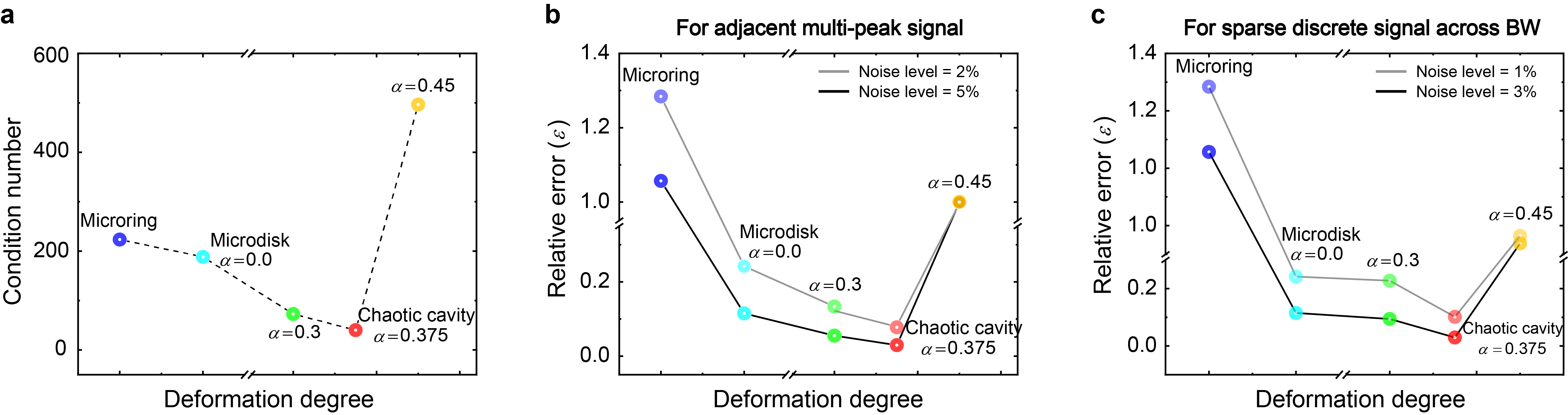


**Fig. S20 |** Characteristic comparison of response matrices for microring, microdisk, and chaotic cavities with *α* = 0.3, 0.375 (optimal chaotic cavity), and 0.45. **a,** Condition numbers. **B,** Relative errors for the closely spaced multi-peak discrete signal with different noise levels. **c,** Relative errors for a sparse discrete signal with varying peak intensities across the bandwidth, with different noise levels.

**S14. More continuous signal reconstruction and analysis**

Fig. **S21** illustrates the reconstruction of the continuous bandpass spectral with a low relative error of 0.121. This bandpass signal is generated by an EDFA and coded by a commercial waveshaper filter (Finisar 1000s).


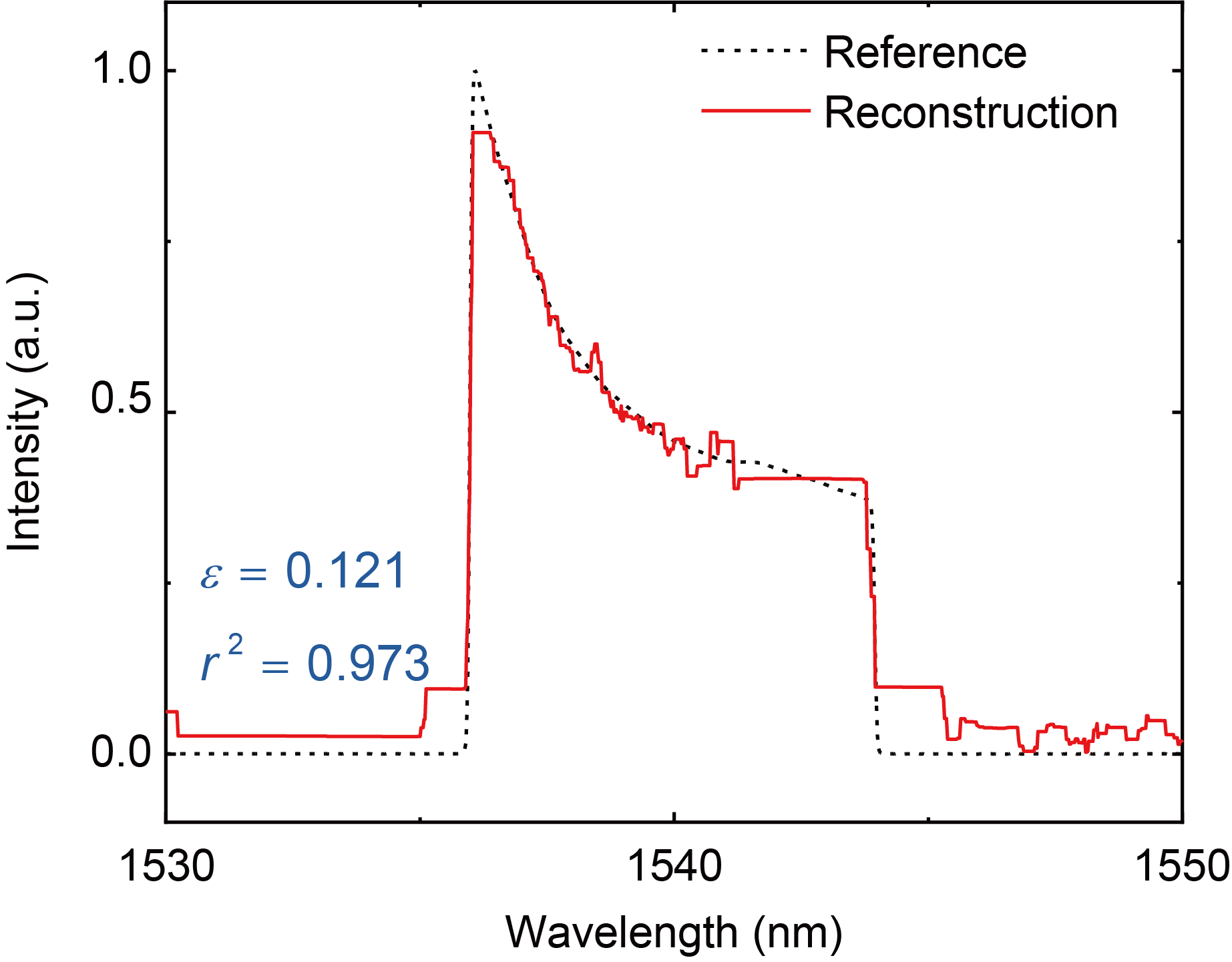


**Fig. S21 |** Reconstruction result of continuous bandpass spectral signal.

Furthermore, we also tested the resolvability of our chaos-assisted spectrometer for hybrid spectra, with different wavelengths of a laser peak combining a smooth continuous Gaussian signal spectra that generated from an EDFA source and filtered by a waveshaper (Finisar 1000s). The resolved spectra are illustrated in Fig. **S22a** and **b**, with low relative errors (ε) of 0.120 and 0.118, respectively.


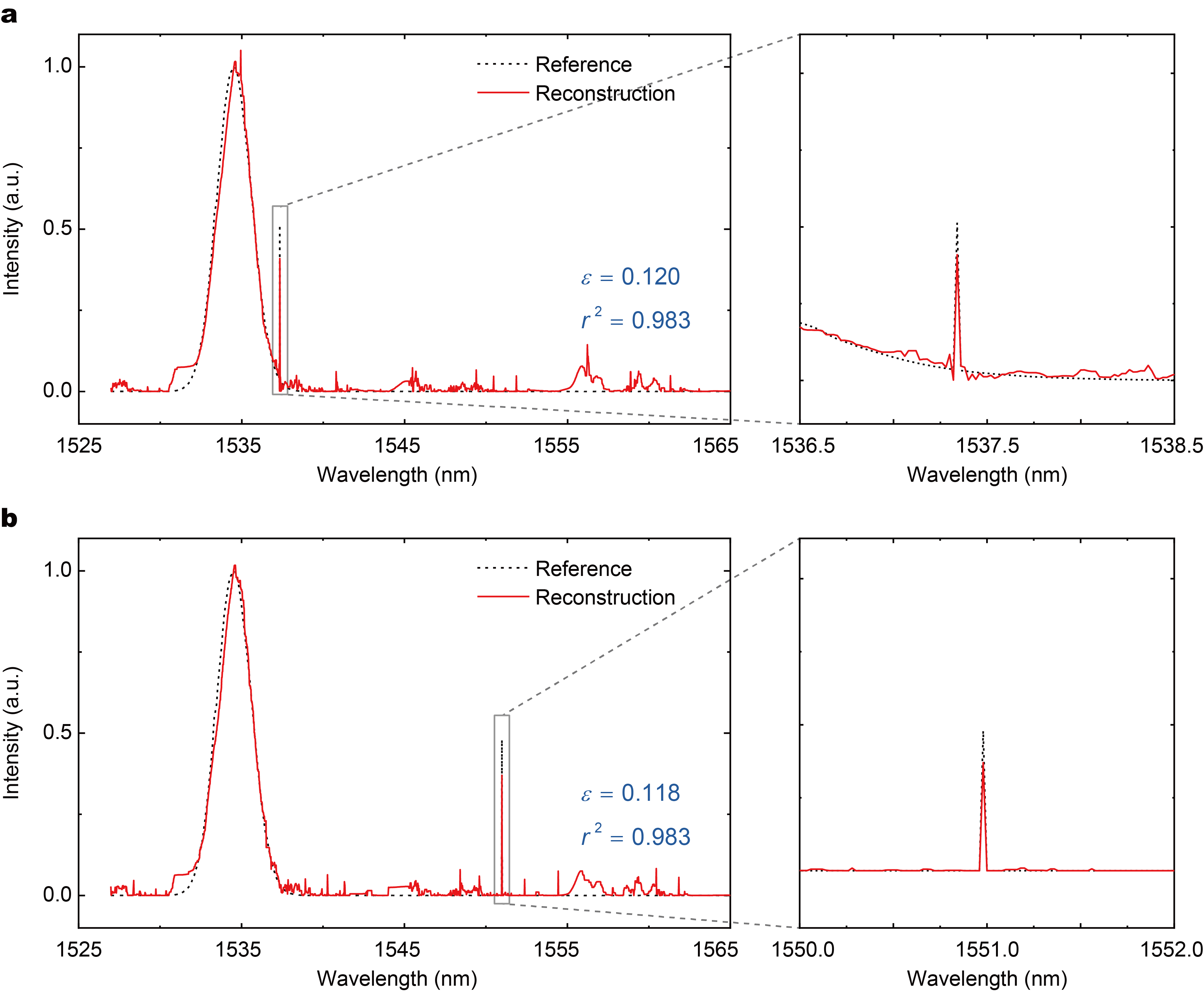


**Fig. S22 |** Reconstruction results of hybrid spectra encompassing both smooth continuous and narrowband spectral components, with the single-peak laser signal located in different positions, **a** and **b**.

**S15. Error analysis for the reconstruction of Continuous signals**

We acknowledge that we encounter more challenges in resolving smooth continuous signals compared to the reconstruction of sparse discrete signals. Theoretical analysis of the factors influencing the accuracy will be provided from the following perspectives.

**Continuous signals reconstruction error analysis**

First, in our miniaturized chaos-assisted spectrometer, compressed sensing is employed. As summarized in Supplementary Information **S19**, we have achieved a fairly high compression ratio among all on-chip computational spectrometers. A core principle of compressed sensing is sparsity. This refers to the property where a signal is represented by a sparse set of coefficients in a transform domain, with the majority being zero or nearly zero, which means signals can be represented by only a few significant coefficients. Relying on the sparsity, compressed sensing allows for significant matrix dimension reduction without losing information content, facilitating efficient signal reconstruction with fewer sampling channels.

However, challenges arise when compressed sensing is applied to continuous smooth signals that contain a large amount of uniformly distributed non-zero frequency components. Due to the dissatisfaction with sparsity, information loss occurs under a lower sampling rate, especially under high compression ratio conditions. To address this, one might consider increasing sampling channels or incorporating more prior information and constraints, which necessitates more sophisticated algorithms and complicates parameter tuning. This is the intrinsic reason why continuous signals are more difficult to recover compared to discrete signals using reconstruction algorithms. This can be validated by the numerical calculation simulation presented in Fig. **S14** of Supplementary Information **S11**. Even without regularization, discrete signals can be resolved with great accuracy of minimal error. However, the response matrix becomes incapable of resolving smooth and stepped continuous signals, which are completely misinterpreted. Furthermore, the absence of an active optical bench along with a pneumatic system, and a temperature controller also introduces extra measurement noises.

Next, consider the discretization process. Our miniaturized chaos-assisted spectrometer yields widely distributed sharp resonance peaks with high Q-factors, exhibiting significant variations around the resonant wavelengths. The drastic changes in the top of resonance peaks are challenging to capture when sampling the transmissions with large wavelength grids. We experimentally validate this by comparing the measured transmission spectra of the same chaos-assisted spectrometer device under two different wavelength sampling grids: 1 pm and 10 pm, as shown in Fig. **S23**. A coarser wavelength grid cannot effectively capture the sharp transmission peaks, resulting in measurements that deviate from the ground-truth transmission properties of the device. The differences in transmission and FWHM under two different wavelength sampling grids are labeled in Fig. **S23**. This discrepancy can lead to large errors when using the pre-calibrated responses sampled with a coarse wavelength grid (like 10 pm) to resolve the collected output power **S**, because **S** is the spectral response that is transmitted and filtered by the ground-truth transmissions of the chaos-assisted spectrometer.

In fact, this drawback is inherently due to the high Q-factor of the device and becomes more pronounced in the response matrices with sharper optical features and drastic changes, which are essential for achieving high reconstruction resolution. Essentially, there exists a trade-off between spectral resolution and solvability for continuous signals. By adapting a response matrix with smoother features with less fluctuation, while sacrificing spectral resolution, helps to minimize this discretization error and delivers more accurate solutions for continuous signals. This trade-off can be mitigated by increasing the number of sampling points with a finer wavelength grid and increasing sampling channels simultaneously, although this significantly increases calibration time, computational demands, and the complexity of the solution process.


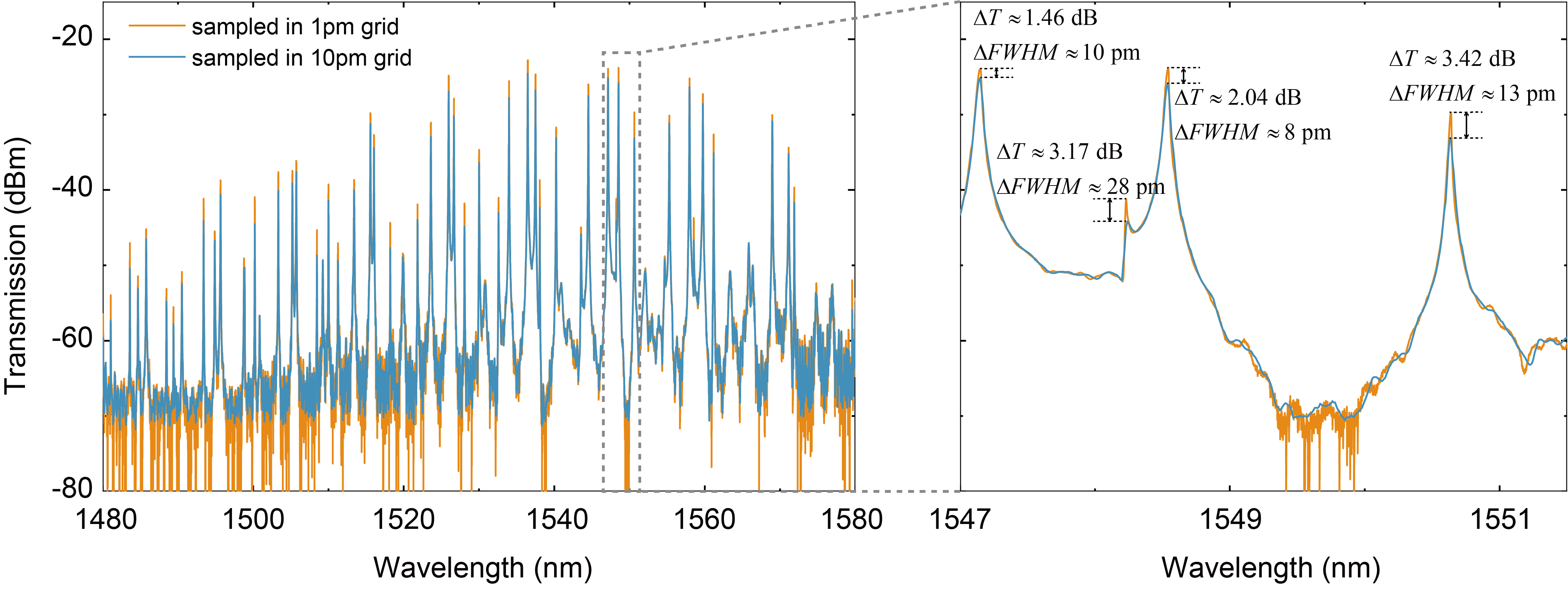
**Fig. S23 |** Transmission spectra of the same chaos-assisted spectrometer when sampled with 1 pm and 10 pm wavelength grid, respectively. Insets show some zoom-in transmissions that cannot be correctly captured under a coarse wavelength grid, as labeled by black dashed squares.

**S16. Dynamic range**

The dual-peak discrete signal, produced by two CW lasers and combined using a 3-dB coupler, consists of one peak in the central wavelength region and another in the longer wavelength region. To further validate the reconstruction capability, the latter peak is further attenuated by an optical attenuator to an intensity that is 13 dB lower than the signal at the center of the wavelength range. As depicted in Fig. **S24**, despite the significantly lower intensity of the peak in the longer wavelength region, reconstruction accuracy for this signal is high with a relative error of only 0.057. Due to the effect of noise, the peak signal-to-noise ratio is approximately 15 dB, and the weaker peak intensity is only 2 dB above the noise level, which limits further testing for dynamic range.


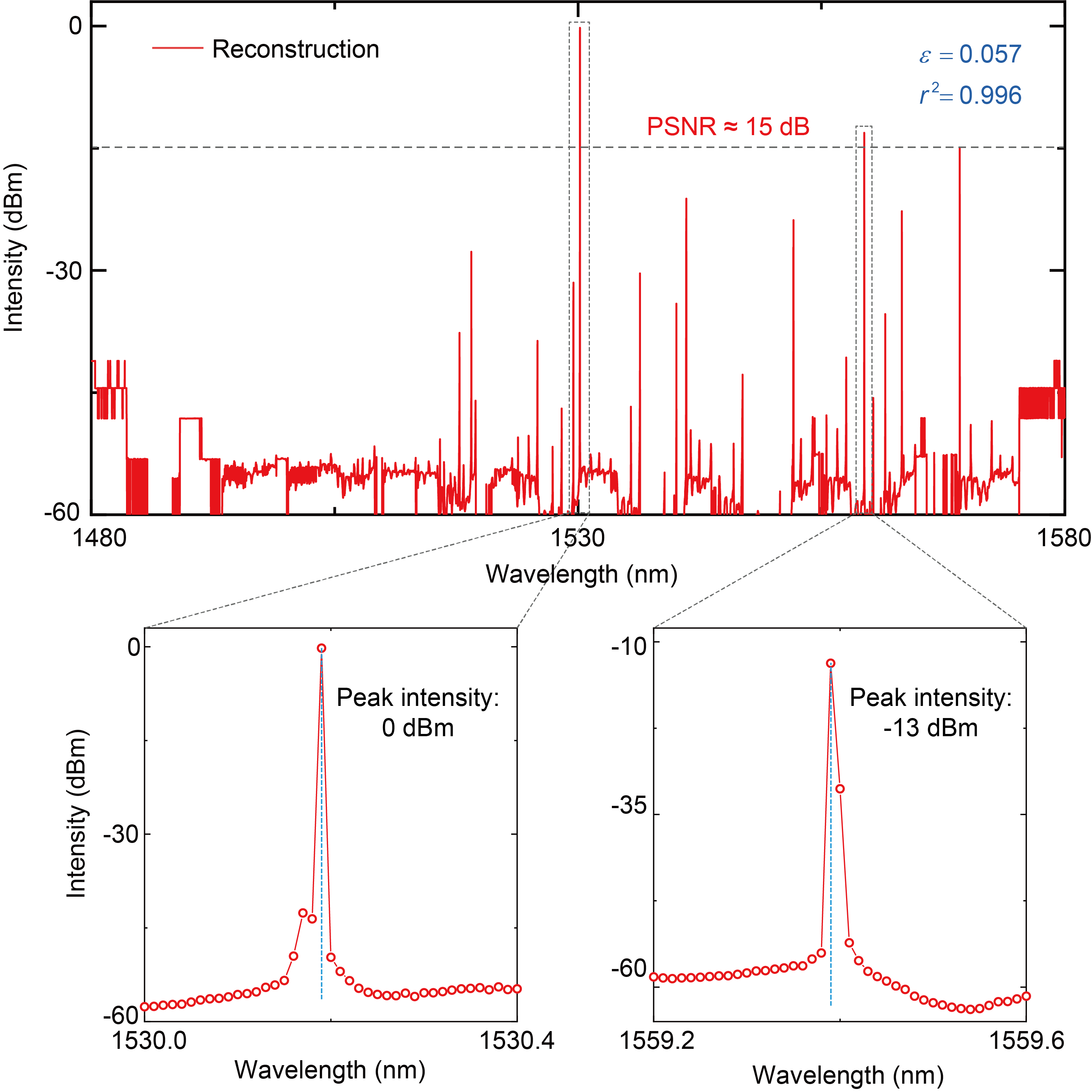


**Fig. S24 |** Experimental reconstruction result of dual-peak signals with significant intensity disparities. Reconstructed spectrum is plotted in red dots and lines, while blue dashed lines indicate the reference incident signal.

**S17. Stability**

**Electrical driving fluctuations test**

We conducted experimental tests on electrical driving fluctuations. We repeat the calibration/measurement process for the chaos-assisted spectrometer, detailed in the Methods section. A linearly swept external driving voltage sequence, generated by a Keithley 2400 source meter and programmed via computer, is applied to the Ti heater of the chaos-assisted spectrometer through electrical packaging. The electrical current is monitored by the source meter and recorded in the computer. The transmission spectrum comprising 10,000 wavelength points is collected from the device’s drop port by a power meter under each external power, forming the response matrix. Here, we define “one cycle” as one whole calibration operation in which the heater undergoes a complete heating process from 0 mW to P_max_, taking approximately 750 seconds.

We systematically record the electrical current for each external voltage index (corresponding to each heating channel) of initial operation, as well as following 5, 10, 15, 20, and 25 cycles, and after 1 day and 7 days, respectively, as illustrated in Fig. **S25a**. Insets highlight the zoom-in electrical currents at approximately the 150th and 300th heating channels. The mean squared errors (MSEs) between the electrical currents measured after multiple cycles/days compared to the initial measurement are summarized in Table. **S3**, maintaining at an extremely low level, with MSE calculations based on current values in milliamperes. Fig. **S25b** provides a direct illustration of the current fluctuations, which are observed to be minimal, at approximately 0.1% of the current value. In addition, the Variance (*σ*^2^) in current values of these operations at each heating power is detailed in Fig. **S25c**, indicating strong consistency of electrical properties across cycles or days.

**Table. S3.** MSEs between electrical current values after multiple cycles/days and initial measurement.

| After | 5 cycles | 10 cycles | 15 cycles | 20 cycles | 25 cycles | 1 day | 7 days |
| --- | --- | --- | --- | --- | --- | --- | --- |
| MSEs | 1.288×10^−5^ | 7.053×10^−5^ | 3.983×10^−5^ | 2.358×10^−5^ | 6.769×10^−5^ | 1.168×10^−4^ | 1.052×10^−4^ |

Furthermore, we conducted tests using an additional spectrometer device located differently on the chip to further evaluate the electrical stability of our chip and packaging, as presented in Fig. **S25d** to **S25f**. The results demonstrate similar slight to negligible electrical fluctuations across operations. Based on the above demonstrations, we claim that the electrical properties of our chaos-assisted spectrometer systems—including the fabrication quality of the Ti heaters and metal interconnection layer, as well as the electrical packaging—exhibit robust stability and reliability, thereby supporting the feasibility of reconstruction.


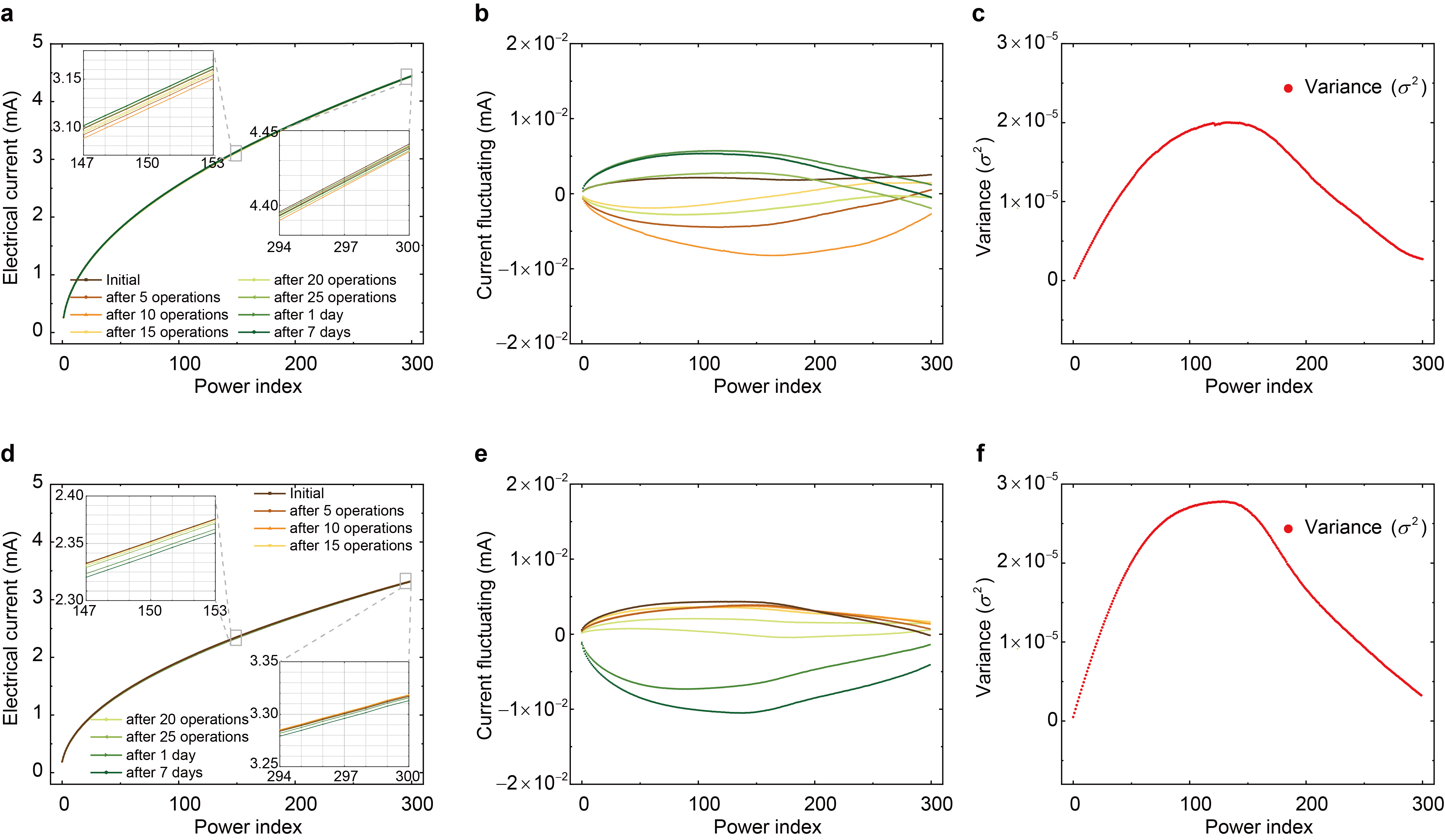


**Fig. S25 |** Electrical property measurement for two chaos-assisted spectrometer devices located differently on the fabricated chip, displayed separately in the top and bottom rows. **a** and **d**, Electrical current value under each heating power of initial measurement, after 5, 10, 15, 20, 25 cycles, after 1 day, and after 7 days, respectively. **b** and **e**, Electrical current fluctuations across these operations. **c** and **f**, Calculated Variance across these operations at each heating power.

**Temperature effect analysis**

Optical package, wire bonding, and electrical package are utilized to eliminate current fluctuating and spatial oscillation of multi-axis optical systems. However, we didn’t incorporate temperature controller systems in our taped-out chip. Consequently, experimentally validating the impact of temperature fluctuations on the fabricated spectrometer devices by precisely controlling the temperature is now infeasible. Thus, we present simulation results for our miniaturized chaos-assisted spectrometer, obtained via 3D-FDFD analysis, to elucidate temperature sensitivity, as the thermos-optic effect for silicon has been widely studied. The transmissions under different temperatures are given in Fig. **S26**. With the temperature rising, the whole simulated transmission spectra would occur a small spectrum redshift. The insets illustrate that our device experiences a redshift of 0.4 nm under a temperature rising of 5K. Consequently, the device’s redshift rate is 80 pm/degree. Accordingly, considering the spectra analysis performance of our chaos-assisted spectrometer, we estimate the thermal stability in our device is about ±0.25℃. We also summarize the thermal stability performance of some reported computational spectrometers in Table. **S4**. Honestly, the thermal stability performance of our device is indeed lower than some previous works, where the main reason is the relatively high thermos-optic coefficient of silicon. To enhance the divergence of the different heating channels, the chaos-assisted spectrometer is demonstrated in a silicon platform for sufficient decorrelation. Therefore, the influence of temperature tremble in our chaos-assisted spectrometer is difficult to avoid. The thermal stability of our chaos-assisted spectrometer just exposes the moderate performance compared to the reported computational spectrometers.


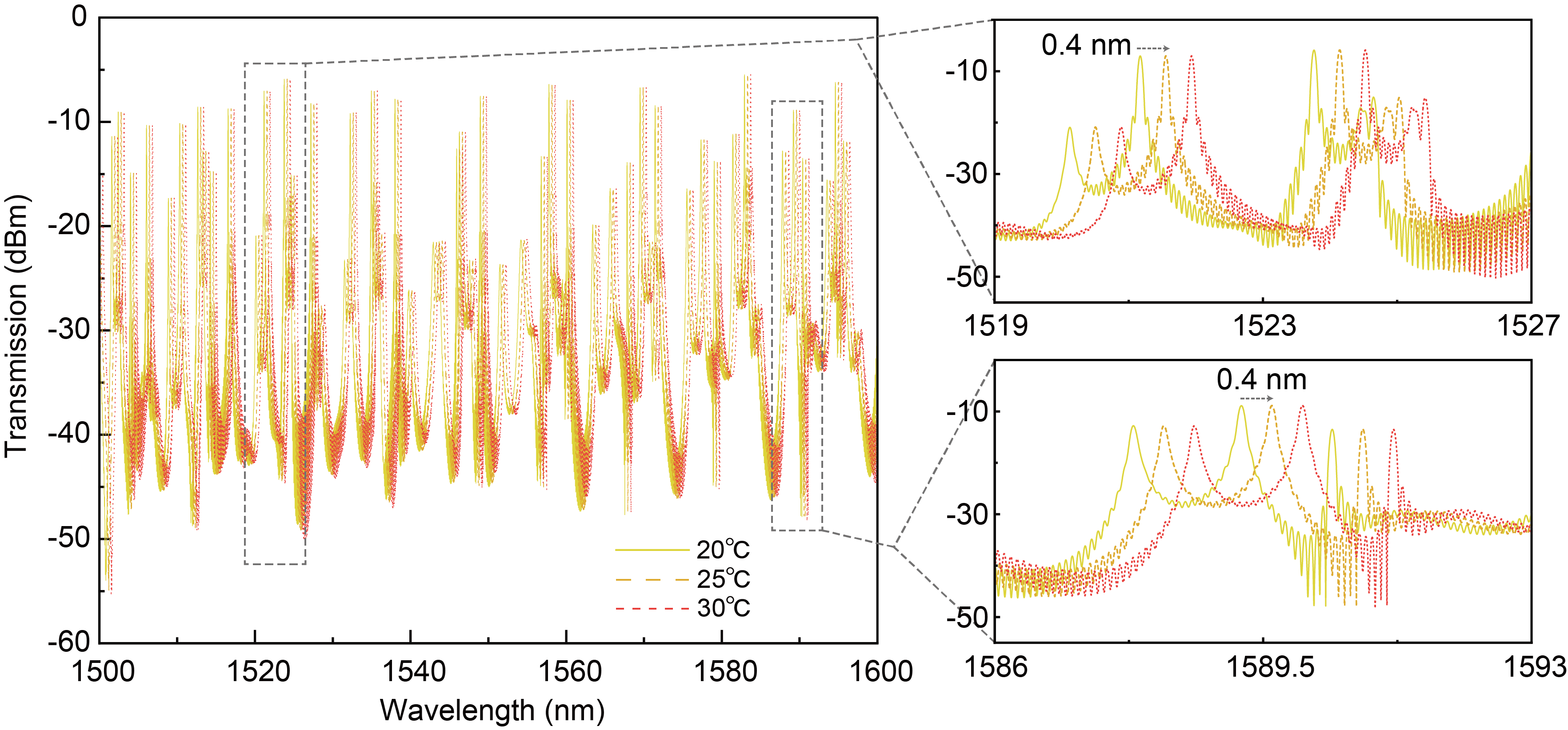


**Fig. S26 |** Transmission spectra of one sampling channel under different temperatures of 20℃, 25℃, and 30℃. The insets exhibit a small spectrum redshift with the temperature rising.

**Table. S4.** Performance comparison of thermal stability for some reported computational spectrometers.

| **Ref** | **Scheme** | **Footprint**  **(μm^2^)** | **Resolution**  **(nm)** | **Bandwidth**  **(nm)** | **Thermal stability** |
| --- | --- | --- | --- | --- | --- |
| Xu. et al.^10^ | Multimode ring | 700 | 0.08 | 100 | ±0.014℃ |
| Sun. et al.^11^ | Microdisk | 40000 | 0.2 | 20 | ~±0.12℃ |
| Redding. et al.^12^ | Disorder media | 1250 | 0.75 | 25 | ±4℃ |
| Zheng. et al.^13^ | AWG + ring | 9000000 | 0.1 | 27 | ~±0.67℃ |
| Yao. et al.^4^ | Reconfigurable photonics | 4161000 | 0.03 | 115 | ±2℃ |
| Yao. et al.^5^ | Programmable photonics | 7030000 | 0.01 | 200 | ±0.9℃ |
| **This work** | **Chaotic cavity** | **440** | **0.01** | **100** | **~±0.25℃** |

In addition, our experiments are conducted in a relatively enclosed laboratory maintained at a nearly constant temperature, equipped with an air conditioning system that remains consistently operational. Therefore, the experiments under the operational temperature can be guaranteed.

**Overall stability test**

Taking into account all potential factors generating measurement noise, including electrical fluctuations, temperature variations that cannot be independently tested, spacial oscillations of the testing system, the optical bench, etc, inherent white noise, and so on, we conduct the calibration process for the chaos-assisted spectrometer device to obtain complete response matrices initially, after 20 cycles, after 1 day and after 7 days, for further validating the overall stability. The calibration process is detailed in the Methods section. The response matrix that was initially measured, recorded as $\mathbf{T}_{\mathrm{initial}}$ is exhibited in Fig. **S27a**. For the response matrices obtained after 20 cycles, as well as after 1 day and 7 days, we calculate the differences between each of them and the initial matrix, denoted as $\Delta\mathbf{T}=\mathbf{T}_{\text{initial}}-\mathbf{T}_{\text{after 20 cy}\text{cl}\text{es}}$, $\Delta\mathbf{T}=\mathbf{T}_{\text{initial}}-\mathbf{T}_{\text{after }\text{1 day}}$, and $\Delta\mathbf{T}=\mathbf{T}_{\text{initial}}-\mathbf{T}_{\text{after }\text{7 days}}$, respectively, as depicted in Fig. **S27b**. Here, we define “one cycle” as a complete calibration operation, taking approximately 750 seconds. To quantitatively assess the similarity among these matrices, we computed the relative error, defined as

$$\frac{\left\| \mathbf{T}_{\mathrm{after}}-\mathbf{T}_{\mathrm{initial}} \right\|_{2}}{\left\| \mathbf{T}_{\mathrm{initial}} \right\|_{2}}$$

and Pearson correlation coefficient (*corr*) to evaluate the linear correlation between these matrices. The computed relative errors and Pearson correlation coefficients for each pair of matrices are presented in Fig. **S27c** and **d**. The analysis yielded an average relative error of 0.0163 and an average Pearson correlation coefficient of 0.996, indicating a remarkably high level of consistency among these matrices.


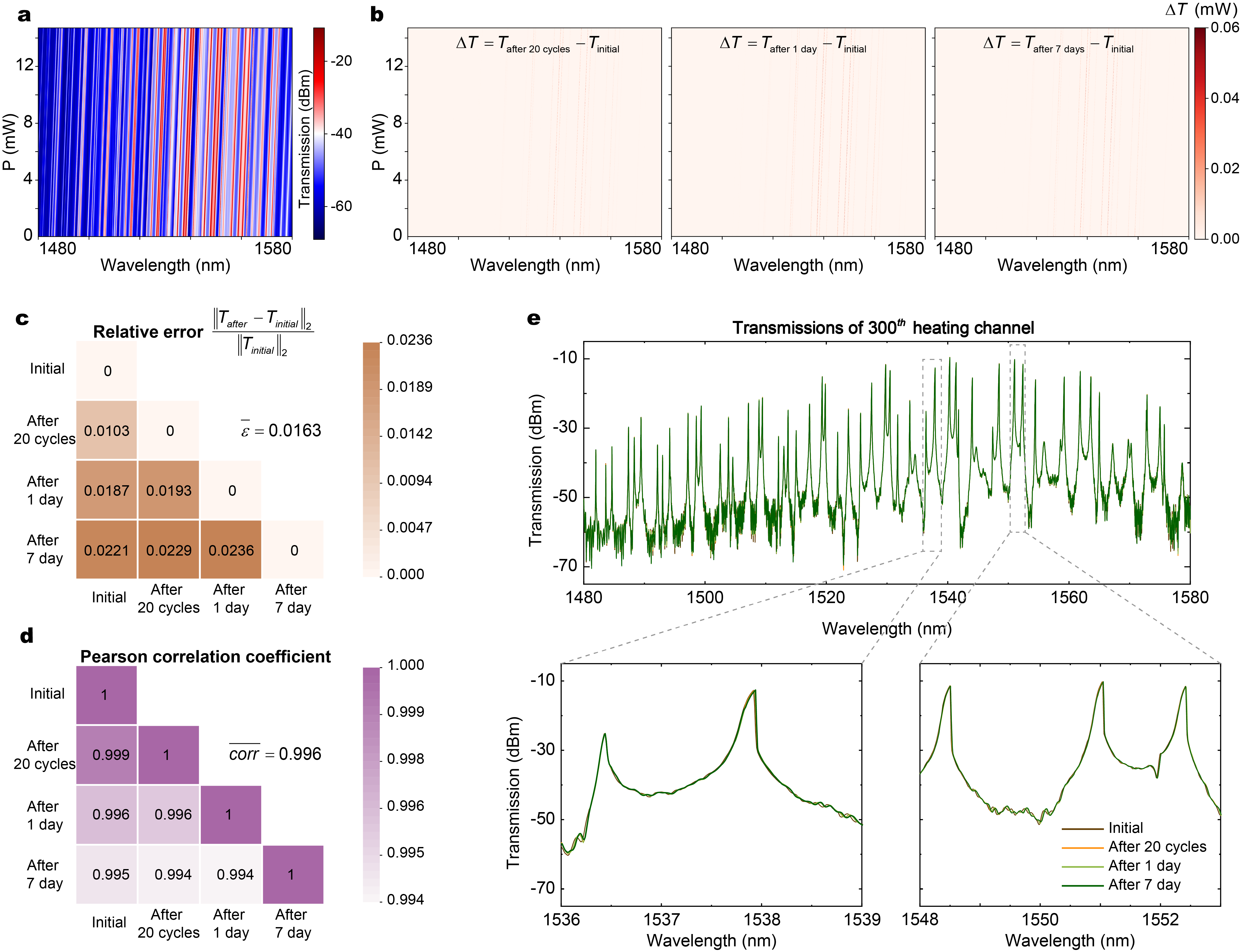


**Fig. S27 |** **a,** $\mathbf{T}_{\mathrm{initial}}$. **B,** The differences between each response matrix obtained after 20 cycles, as well as after 1 day and 7 days and the initial matrix. Similarity among these matrices is quantified by **c,** Relative error, and **d,** Pearson correlation coefficient. **e,** Transmissions of the final heating channel of initial measurement, after 20 cycles, after 1 day and 7 days, respectively.

To provide an intuitive representation of stability across operational cycles spanning several days, we extract transmissions under the last heating channel (300th channel) of the initial matrix, as well as after 20 cycles, 1 day, and 7 days, as depicted in Fig. **S27e**. The selection of the 300^th^ heating channel is warranted, given that the device has undergone a complete heating and optical data collection process, thereby reflecting its final state after an entire operational cycle. The transmission profiles exhibit an almost complete overlap, suggesting a high level of consistency. All quantitative metrics discussed above substantiate the strong consistency of the chaos-assisted spectrometer system’s performance across multiple cycles spanning large temporal intervals, thereby reinforcing the reliability of the obtained measurements and underscoring the robustness and stability of the system’s operational integrity.

Additionally, we experimentally performed reconstruction measurements of a single peak signal after 20 cycles, as well as after 1 day and 7 days, with 1 pm wavelength sampling grid, depicted in Fig. **S28**. Slight picometer-scale shifts in the resolved wavelength are observed. Notably, we opted not to include a temperature controller. The minor discrepancies between initial and subsequent peak reconstructions can be attributed to ambient temperature variations in the lab. Luckily, the impact of picometer scale remains minimal to negligible for most application scenarios. Furthermore, these can be compensated for with a temperature controller or through recalibration of the response matrix under different ambient conditions.


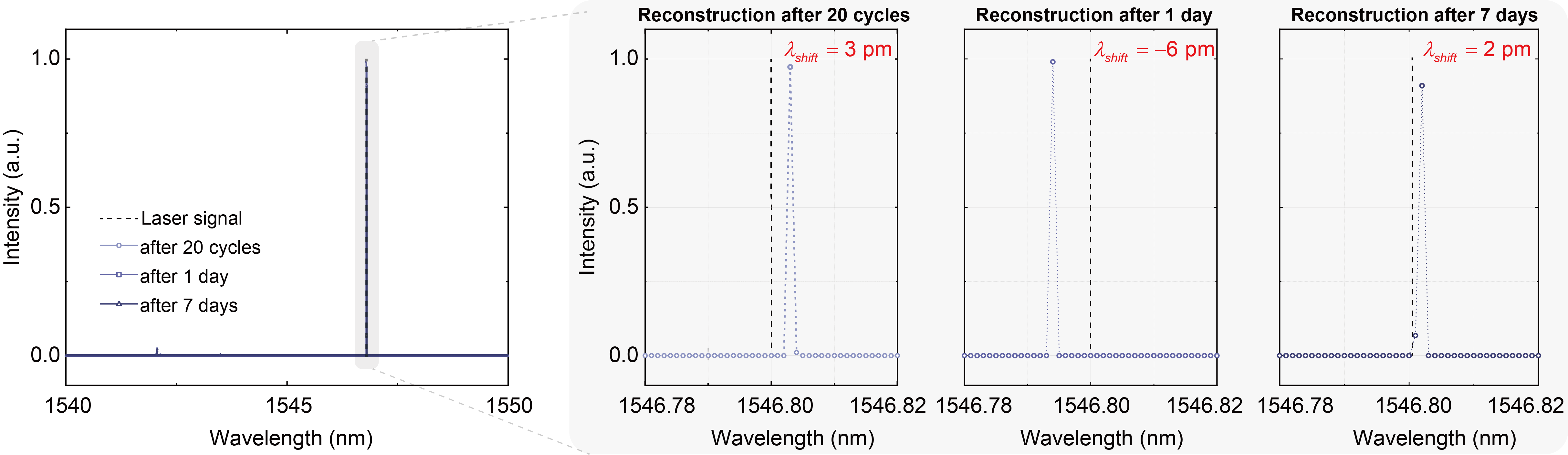


**Fig. S28 |** Experimental reconstruction test of stability. Reconstruction of a single-peak signal after 20 cycles, 1 day, and 7 days.

**S18. Performance comparison for reported computational spectrometers**

| **Method** | **Footprint**  **(**$\boldsymbol{\mu m}^{\boldsymbol{2}}$**)** | **Spatial channels** | **Resolution**  **(nm)** | **Bandwidth (nm)** | **BRR** | **BRFR**  **(μm^-2^)** |
| --- | --- | --- | --- | --- | --- | --- |
| Nanowire^7^ | 0.5×75 | 1 | 10 | 130 | 13 | 3.47×10^-1^ |
| Van der Waals junction^8^ | 22×8 | 1 | 3 | 440 | 146.7 | 8.33×10^-1^ |
| Scattering multiplexer^14^ | 9.4×14.4 | 10 | 50 | 500 | 10 | 7.39×10^-2^ |
| Random scattering medium^15^ | 30×12.8 | 8 | 0.25 | 30 | 120 | 3.13×10^-1^ |
| Stratified waveguide filters^16^ | 35×260 | 32 | 0.45 | 180 | 400 | 4.40×10^-2^ |
| Black phosphorus photodetector^17^ | 9×16 | 1 | 420 | 7000 | 16.67 | 1.16×10^-1^ |
| Random scattering medium^12^ | 50×25 | 25 | 0.75 | 25 | 33.33 | 2.67×10^-2^ |
| Multimode spiral waveguide^18^ | 500×500 | 40 | 0.01 | 2 | 200 | 8×10^-4^ |
| Random scattering medium^19^ | 200×100 | 13 | 0.3 | 15 | 50 | 2.5×10^-3^ |
| Random scattering medium^20^ | 200×100 | 16 | 0.03 | 15 | 500 | 2.5×10^-2^ |
| Nanobeam Array^21^ | 114×6 | 38 | 5 | 70 | 14 | 2.05×10^-2^ |
| Multimode cavities^22^ | 1500×1000 | 1 | 0.005 | 100 | 20000 | 1.33×10^-1^ |
| MZI+MRR^3^ | 520×220 | 64 | 0.02 | 12 | 600 | 5.24×10^-3^ |
| Cascaded nanobeam^23^ | 18×18 | 3 | 0.32 | 16 | 50 | 1.54×10^-1^ |
| Single microdisk^11^ | 200×200 | 1 | 0.2 | 20 | 100 | 2.5×10^-3^ |
| Cavity-enhanced^24^ | 3.5×10^5^ | 11 | 0.005 | 10 | 2000 | 5.71×10^-3^ |
| Coupled MRR^25^ | 60×60 | 1 | 0.04 | 100 | 2500 | 6.94×10^-1^ |
| DC integrated MRR^10^ | 20×35 | 1 | 0.08 | 100 | 1250 | 1.79 |
| MZI+MRR^4^ | 7300×570 | 4 | 0.03 | 115 | 3833.3 | 9.21×10^-3^ |
| Cascaded MZI^5^ | 1900×3700 | 1 | 0.01 | 200 | 20000 | 2.84×10^-3^ |
| MRR^26^ | 16×16 | 1 | 0.4 | 10 | 25 | 9.77×10^-2^ |
| Van der Waals junction^27^ | 220 | 1 | 5 | 400 | 80 | 3.63×10^-1^ |
| Van der Waals diode^28^ | 30×20 | 1 | 2 | 1100 | 550 | 9.17×10^-1^ |
| Stratified waveguide filters^29^ | 4000000 | 32 | 0.12 | 120 | 1000 | 2.5×10^-4^ |
| Silicon MEMS + Cascaded MZI^30^ | 3810×80 | 1 | 8 | 450 | 56.25 | 1.85×10^-4^ |
| Van der Waals junction^31^ | 25×25 | 1 | 0.35 | 700 | 2000 | 3.2 |
| **Our work** | **20×22** | **1** | **0.01** | **100** | **5000** | **22.73** |

*BRR: Bandwidth to resolution ratio

*BRFR: BRR to footprint ratio


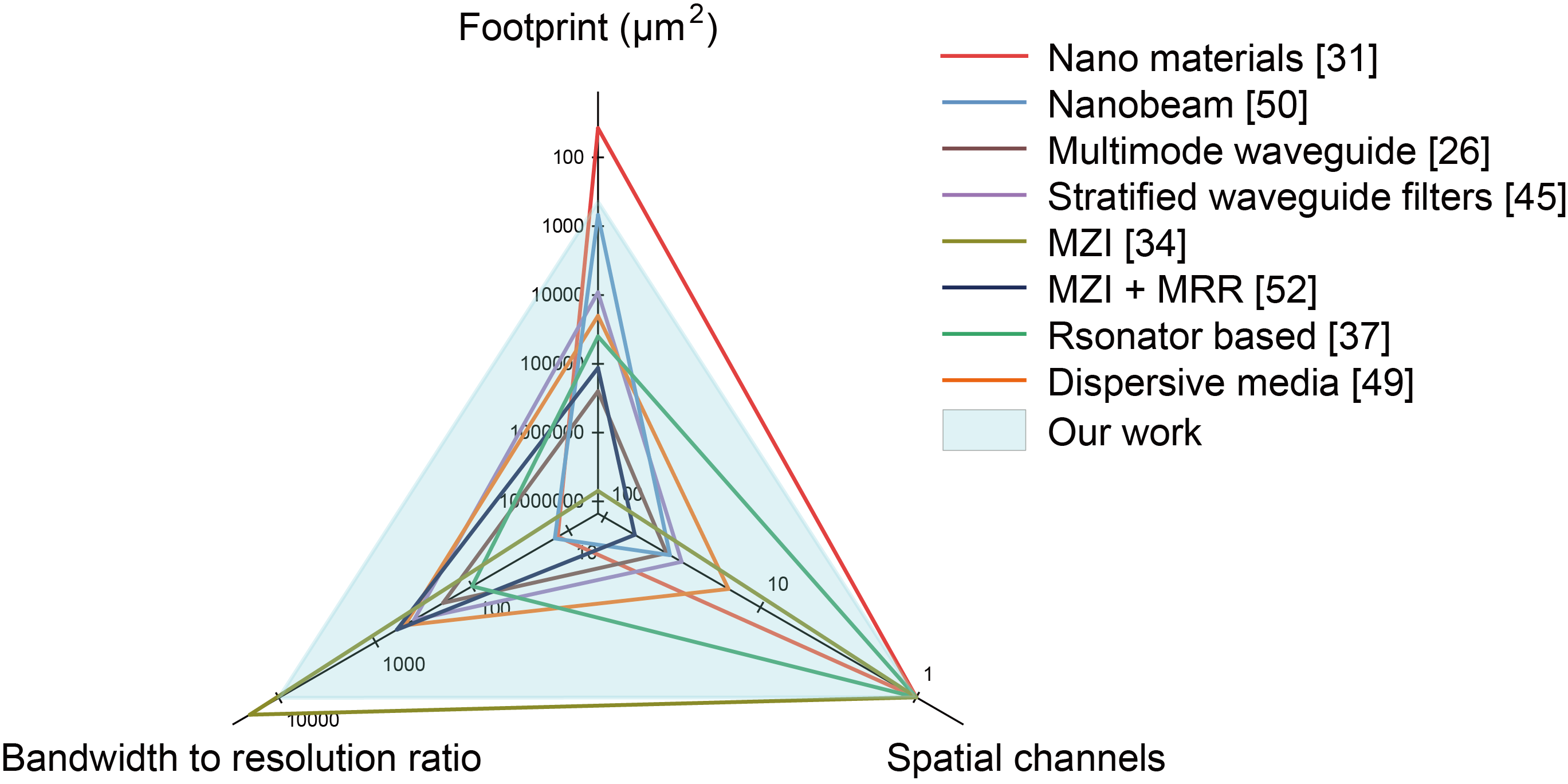


**Fig. S29 |** The performance comparison between our work and other typical computational spectrometers, where MZI and MRR are abbreviations for Mach-Zehnder interferometer and microring-resonator. The marks of reference refer to the reference in the manuscript.

**Table.** **S5**. Power consumption comparisons of the other reported on-chip computational spectrometers.

| **Ref** | **Power Consumption (mW)** |
| --- | --- |
| Zhang. et al.^23^ | 30 |
| Xu. et al.^22^ | 50 |
| Xu. et al.^10^ | 45 |
| Sun. et al.^11^ | 160 |
| Zhang. et al.^24^ | 30 |
| Yao. et al.^5^ | 350 |
| **Our work** | **16.5** |

**S19. Compression ratio comparison**

We have reviewed almost all published works in the field of on-chip computational spectrometers from 2013 to date, summarizing their spatial or temporal channels, resolved spectral channel numbers, as well as the sampling channel tunability in Table. **S6**. Based on the calculated compression ratios presented in the last column, we achieved a fairly high compression ratio among on-chip computational spectrometers. The work with higher compression ratios than ours is highlighted with an orange background.

**Table. S6.** Summarization of the number of spatial/temporal channels, spectral channel, and compression ratio.

|  | **type** | **Spatial channel** | **Temporal channel** | **Spectral channel number** | **Channel tunability** | **Compress-ion ratio** |
| --- | --- | --- | --- | --- | --- | --- |
| Hui Cao^12^ | Random media | 25 |  | 100 | N | 4 |
| Hui Cao^18^ | Multimode wg | 15 |  | 332 | N | 22.13 |
| Tawfique Hasan^7^ | Single nanowire | 38 |  | 140 | N | 3.68 |
| Wolfram Pernice^20^ | Random media | 16 |  | 2000 | N | 125 |
| Wolfram Pernice^19^ | Random media | 13 |  | 400 | N | 30.78 |
| Andrea Fiore^14^ | Designed media | 10 |  | NG | N | NG |
| Koray Aydin^15^ | Random media | 8 |  | 150 | N | 18.75 |
| Yeshaiahu Fainman^16^ | SWF | 32 |  | 450 | N | 14.06 |
| Fengnian Xia^17^ | BP |  | 41 | NG | Y | NG |
| Hon Ki Tsang^3^ | MZI+MRR array | 64 |  | 600 | N | 9.375 |
| Xinliang Zhang^21^ | Nanobeam array | 38 |  | NG | N | NG |
| Xinliang Zhang^23^ | Cascaded nanobeam | 3 | NG | NG | Y | NG |
| Daoxin Dai^24^ | Cascaded MRR | 10 | 9 | 1200 | Y | 13.33 |
| Zhipei Sun^8^ | Van der Waals junction |  | ~100 | 100 | Y | 1 |
| Hon Ki Tsang^25^ | Cascaded MRR |  | 3001 | 3001 | Y | 1 |
| Hon Ki Tsang^22^ | Multimode cavity |  | 20000/2000 | 20000 | Y | 1 or10 |
| Hon Ki Tsang^10^ | Multimode MRR |  | 1250 | 1250 | Y | 1 |
| Richard Penty^4^ | MZI+MRR array |  | 250 | 3833 | Y | 15.3 |
| Richard Penty^5^ | MZI array |  | 729 | 13000 | Y | 17.83 |
| Xuhan Guo^26^ | MRR |  | 300 | 300 | Y | 1 |
| Han-Chun Wu^27^ | Van der Waals junction |  | 9 | 81 | Y | 9 |
| Zhipei Sun^28^ | Van der Waals diode |  | 41 | 31/21 | Y | 0.76 or 0.51 |
| Shilong Pan^29^ | SWF | 64 |  | NG | N | NG |
| Guangya Zhou^32^ | Silicon MEMS |  | 128 | 201 | N | 1.57 |
| Doron Naveh^31^ | Van der Waals junction |  | 2000 | 2000 | Y | 1 |
| **This work** | **Chaotic cavity** |  | **300** | **10000** | **Y** | **33.33** |

*NG: Not given;

*wg: Waveguide;

*SW: Stratified waveguide filters;

*MZI: Mach-Zehnder interferometer;

*MRR: Microring resonator;

*MEMS: Micro-Electro-Mechanical Systems.

**S20. The universality of chaos-assisted spectrometer**

If the chaotic cavity system satisfies the assumption of the ray dynamics model that the size of the proposed chaotic cavity surpasses the limit imposed by the short wavelength of the wave, the chaotic spectra always remain the expected performance. In our preliminary work, we do not assume the materials and wavelength range of our structures but directly exploit the ray dynamics model to analyze the chaotic motion of straight rays in deformed microcavities and optimize the structure parameter of the device. Therefore, the chaotic cavity structure can be universally applicable to various materials and broad wavelength ranges. In our work, the silicon-on-insulator (SOI) platform is utilized for leveraging the high thermo-optic coefficient of silicon (${dn(\mathrm{Si})}/{dT}=1.86\times{10}^{-4}K^{-1}$) in order to distinct different heating channels. The device is operated in the C-band which is widely utilized in the optical communication field. Typically, SOI devices should operate within the bandwidth from 1.2 μm to 4 μm. Here, we further make the simulations for the different operational wavelengths of the chaotic cavity, where the efficient radius of the chaotic cavity, the width of waveguides, and coupling gaps are adjusted in accordance with wavelength. The transmissions and calculated auto-correlation functions are illustrated in Fig. **S30**. In the operational wavelength of 1300 nm and 2000 nm, the chaotic cavity could hold a similar low periodicity level of 0.231 and 0.241, indicating an effective capability to generate quasi-random response matrices.


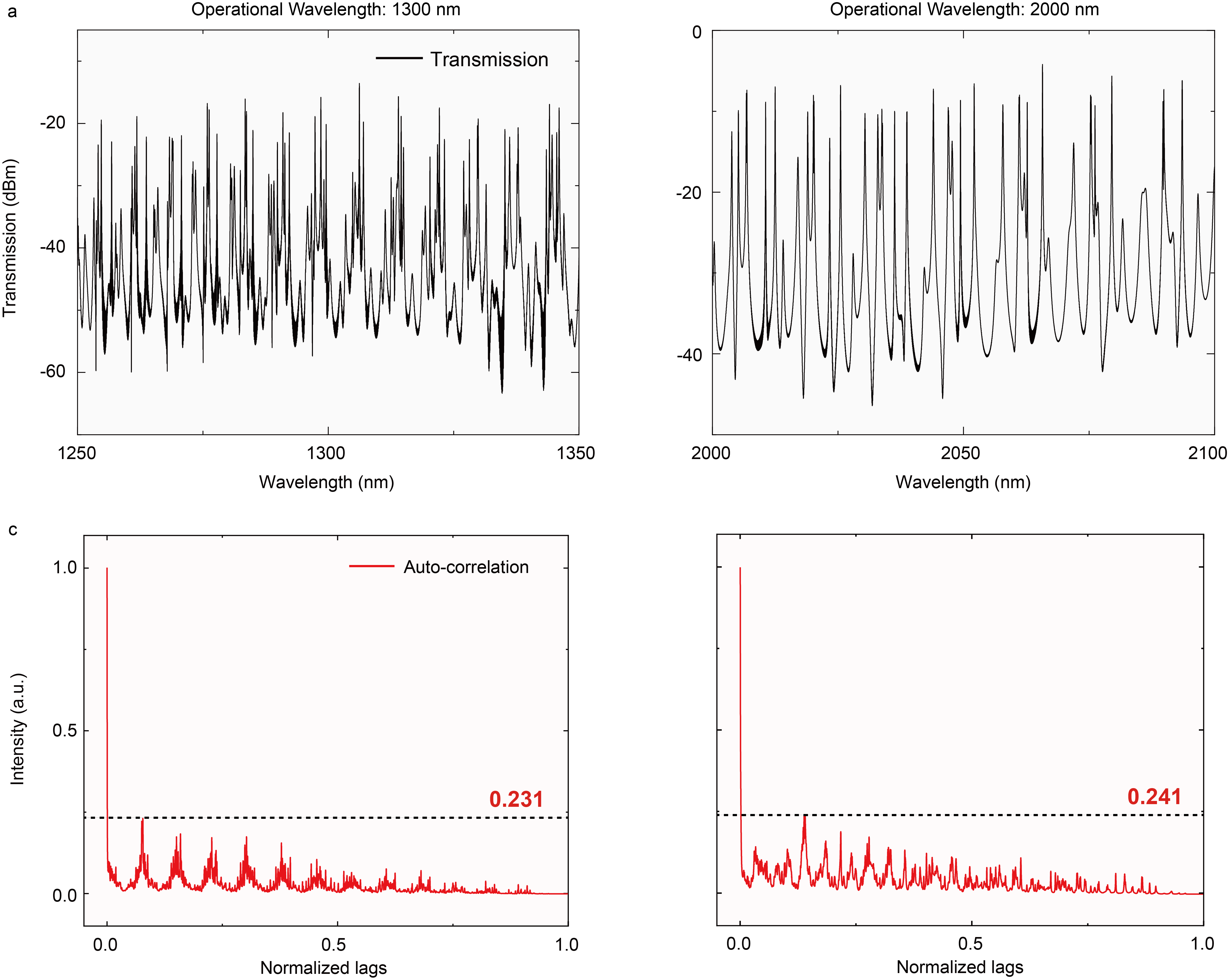


**Fig. S30 |** Transmission and calculated auto-correlation function for the different operational wavelengths of the chaotic cavity.

Additionally, it’s noted that the different platforms, for example, the silicon nitride platform, has the potential to transplant this design into visible light wavelength band hence with broader detectable bandwidth. However, there is a tradeoff regarding to the application of spectrometers. Silicon nitride exhibited one order of magnitude lower thermos-optic coefficients (${dn({Si}_{3}N_{4})}/{dT}=2.45\times{10}^{-5}K^{-1}$) than silicon. Thus, the thermal tuning process in our chaos-assisted spectrometer would consume much higher power. Plus, an insufficient decorrelation between transmissions of heating channels under the same heating power may lead to a “noisy” matrix, indicating strong similarity between each row in the response matrix. Reconstruction accuracy and stability can be largely impaired.

**S21. Miniaturization limitation**

For our chaos-assisted spectrometer, the miniaturization requires to match the used theory for the rationality of theoretical analysis. In the ray dynamics model, a crucial assumption is that the scale of cavity surpasses the limit imposed by the short wavelength of the wave so that we can assume the light is a straight ray and analyze the motion in the cavity. The operational wavelength range of our spectrometer device is under the C-band with a center wavelength of 1550 nm, so the theoretical analysis results based on the ray dynamics model would be unreasonable when the footprint of the chaotic cavity decreases to below the limitation exerted by the wavelength of light. On the other hand, the smaller scale of the cavity wouldn’t sustain the high-order resonant modes, causing the reduction of supported resonant modes in the cavity to reversely increase the periodicity of transmission.

To confirm the miniaturization limit of our chaotic cavity, we measure the transmission of the chaotic cavity with the same deformation parameter α of 0.375 and the different efficient radii of 10 μm and 5 μm and calculate the corresponding auto-correlation function, as illustrated in Fig. **S31**. It’s clearly noted that the transmission of the chaotic cavity with a 10 μm effective radius exposes more complex resonant peaks than the transmission of the chaotic cavity with a 5 μm effective radius in Fig. **S31a** and **S31b**. The periodicity level of the calculated auto-correlation function of the chaotic cavity with a 10 μm effective radius is 0.23 while the periodicity level of the calculated auto-correlation function of the chaotic cavity with a 5 μm effective radius rises to a high value of 0.38, as shown in Fig. **S31c** and **S31d**. Clearly, when the efficient radius continues to decrease to 5 μm, the basic assumption in the ray dynamics model would be broken and the number of supported resonant modes would sharply decline, resulting in the increased periodicity which we don’t expect in this spectrometer. Consequently, we already designed the possible minimum feasible size for the spectrometer system with a compact footprint of 10 μm effective radius.


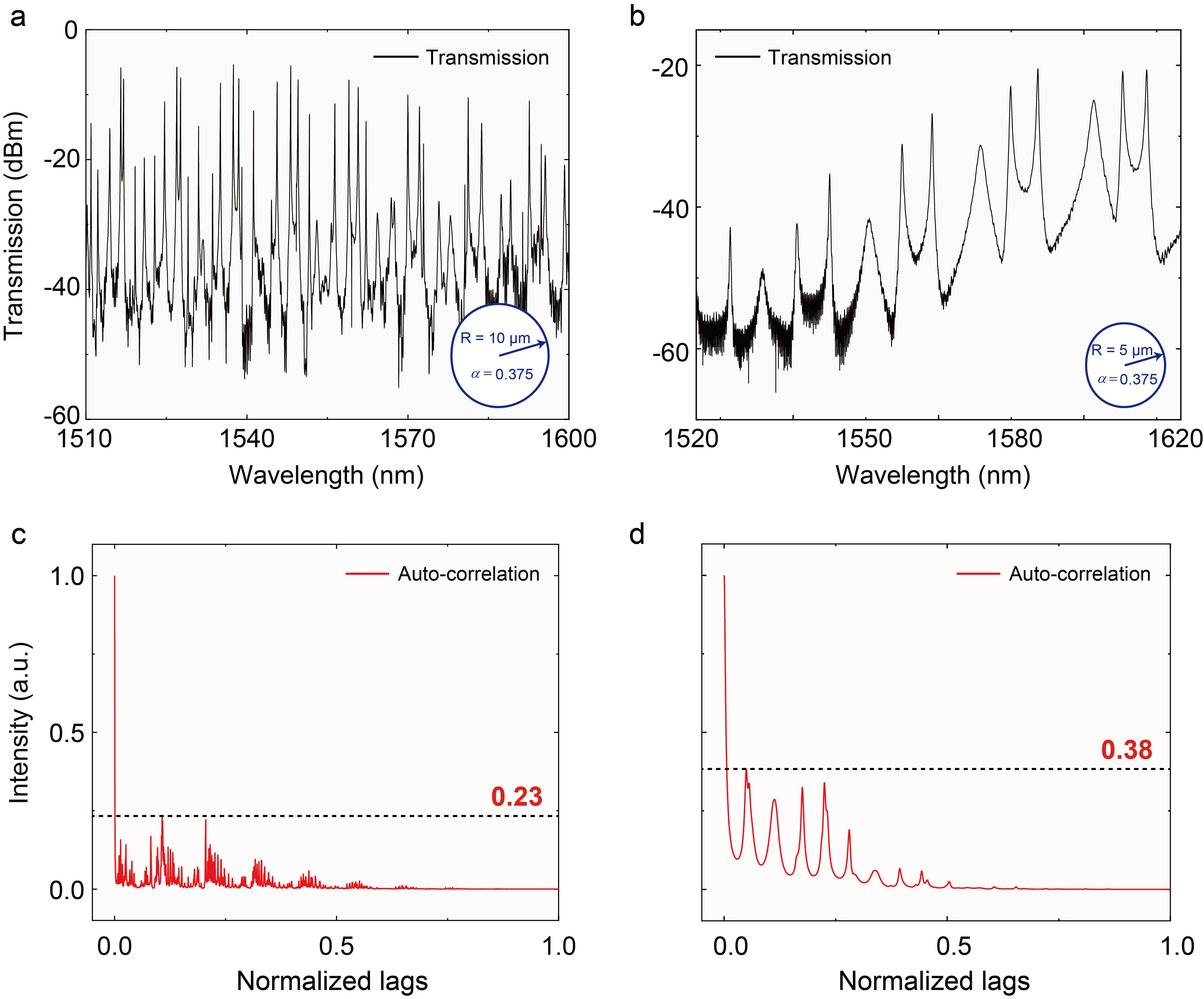


**Fig. S31 | a,** Measured transmission of the chaotic cavity with an effective radius of 10 μm. **b,** Measured transmission of the chaotic cavity with an effective radius of 5 μm. **c,** Calculated auto-correlation function of the chaotic cavity with an effective radius of 10 μm. **d,** Calculated auto-correlation function of the chaotic cavity with an effective radius of 5 μm.

**S22. Reproducibility**

We have fabricated an array of chaos-assisted spectrometer devices on our chip shown in Fig. **S8**, each with varying deformation parameters and coupling gaps. We conducted measurements on a set of spectrometer devices that share the same deformation parameter but feature different coupling gaps. The corresponding transmission spectra and detailed spectral zooms are depicted in Fig. **R32a** and **b**, respectively. In order to facilitate further observation, the transmission spectra were subtly manually shifted to align the resonance wavelengths, as illustrated in Fig. **R32c**. The fundamental resonance mechanism across these devices remains consistent thus the spectral response profiles are essentially congruent, proving that our chaotic cavities can deliver consistent deformation profiles. Minor resonance wavelength shifts are noted, potentially attributable to variations in cavity dimensions arising from nonuniformity in the etching or developing processes. A narrowing of the resonance peaks with increasing gap size is witnessed. This is attributed to the reduction of coupling efficiency associated with the increasing coupling gaps, which enhances *Q*_load_ and consequently narrows the spectral FWHM.


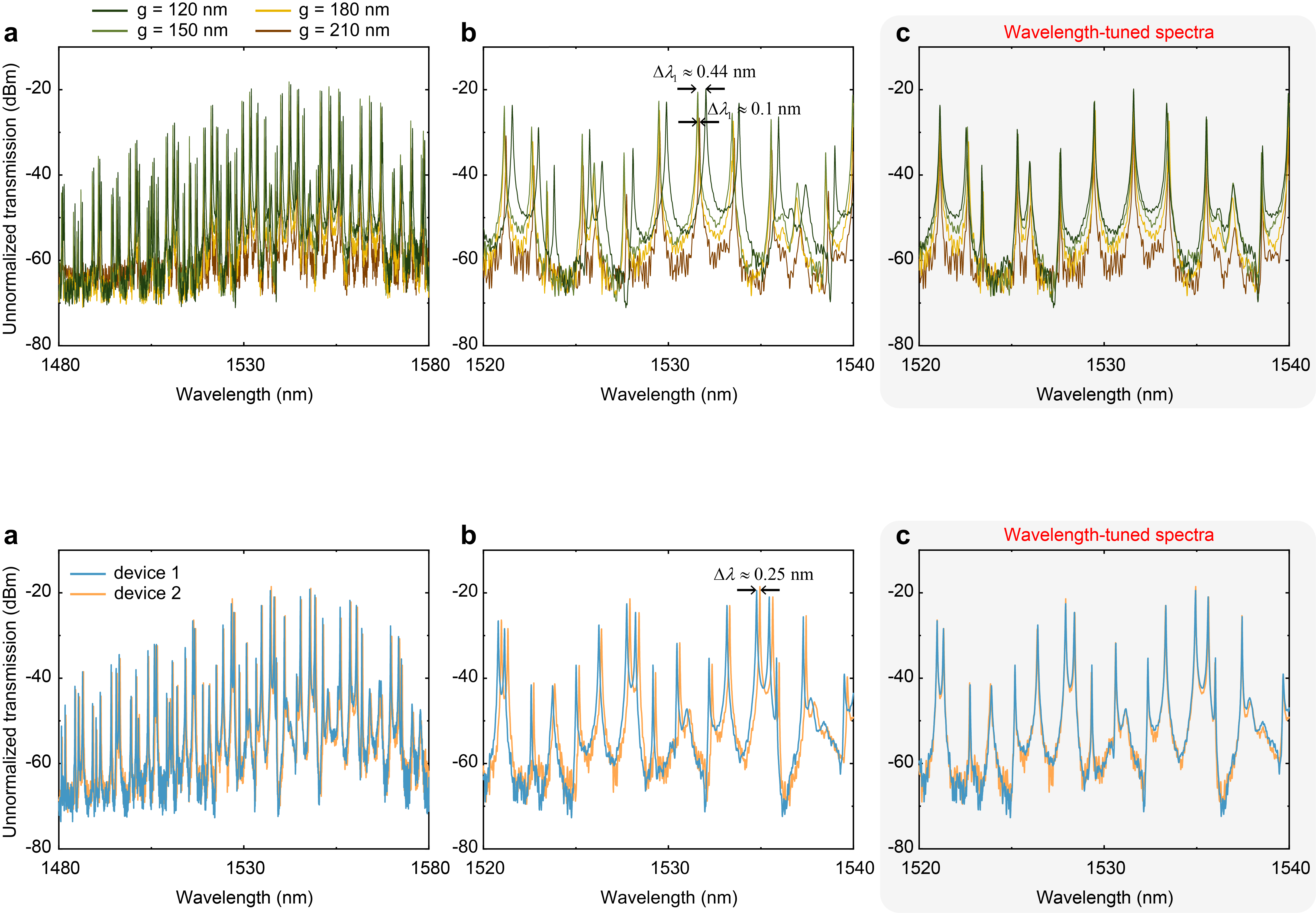


**Fig. S32 | a,** Transmission spectra and **b,** Zoom-in views of four chaos-assisted spectrometer devices with the same deformation parameter and varying coupling gaps on our fabricated chip. **c,** False transmission spectra of the four devices following a minor wavelength realignment, just for enhanced observation and comparative analysis.

In an additional fabricated chip, we identified a pair of identical chaos-assisted spectrometer devices exhibiting the same deformation parameter (*α* = 0.375) and coupling gaps (150 nm), located at different positions on the chip. The transmission spectra, along with detailed zoom-in views, are depicted in Fig. **R33a** and **b**. A slight wavelength shift of approximately 0.25 nm is noted between the two devices. In order to facilitate further observation, the transmission spectra were subtly manually shifted to align the resonance wavelengths, as illustrated in Fig. **R33c**. It is evident that, aside from a minor wavelength shift attributable to fabrication, nearly indistinguishable spectral profiles that align closely are observed. This further validates that our chaotic cavities can deliver consistent deformation profiles after fabrication.


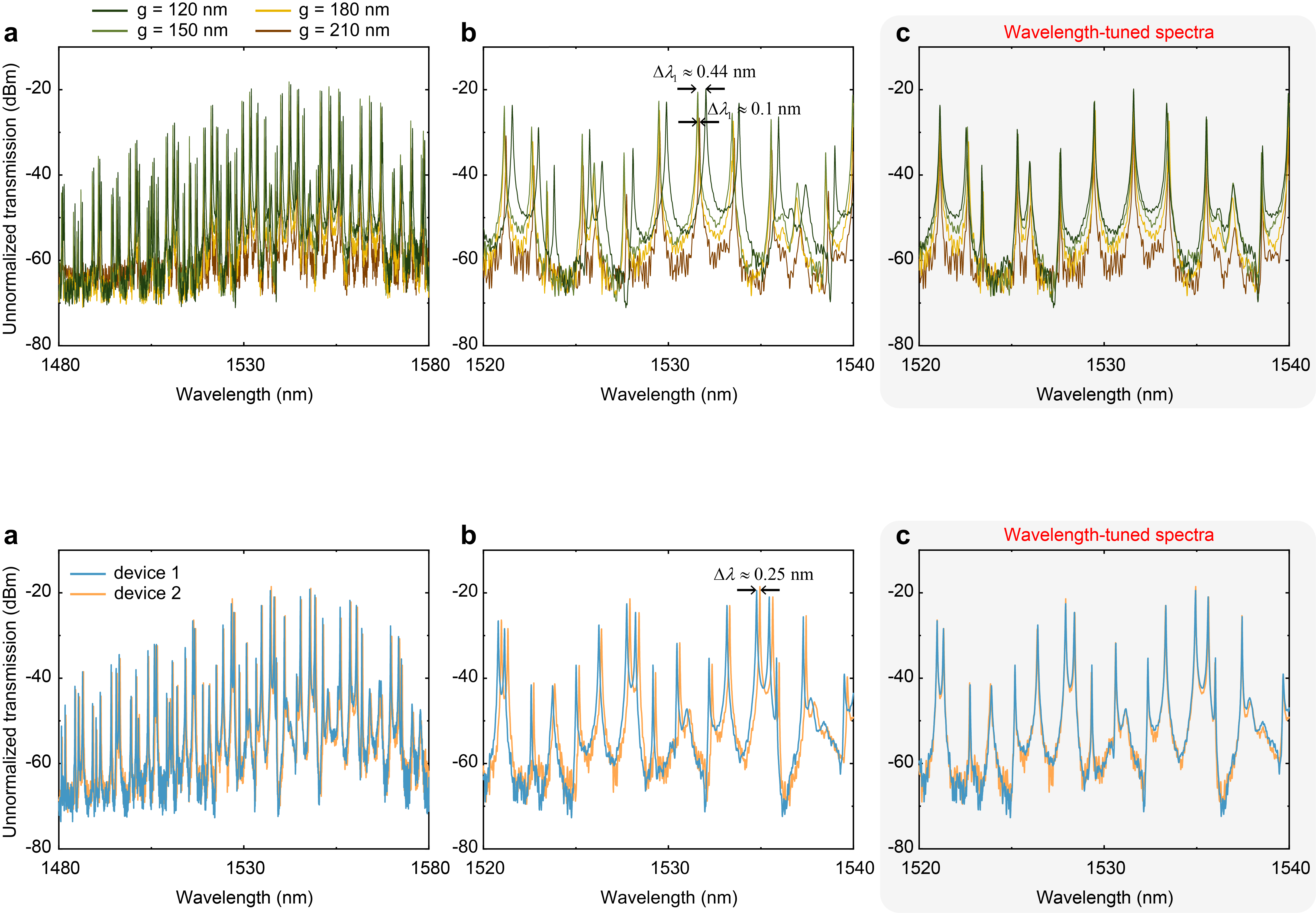


**Fig. S33 | a,** Transmission spectra and **b,** Zoom-in views of two identical chaos-assisted spectrometer devices with the same deformation parameter and the same coupling gaps on another fabricated chip. **c,** False transmission spectra following a minor wavelength realignment, just for enhanced observation and comparative analysis.

The spiral Titanium heater atop the chaotic cavity is utilized to cover nearly all of the chaotic cavity region. The integration of the Ti heater allows us to drive all-supported resonance modes to generate wavelength red-shifting through TO effect. Due to the different mode distributions of different resonant modes in chaotic cavity, each exhibiting varying thermos-optic sensitivity, we summarize the tuning efficiency of these modes with respect to wavelength as plotted in Fig. **S34**. The overall tuning efficiency is estimated to be 203.2 pm/mW. During the potential commercial deployment, the occurrence of slight wavelength shifts can be uniformly corrected by heating the Ti heater with an external power through TO effect, aligning the resonant wavelengths of various devices integrated on the chip. Or more directly, a calibration process can be implemented for each device.


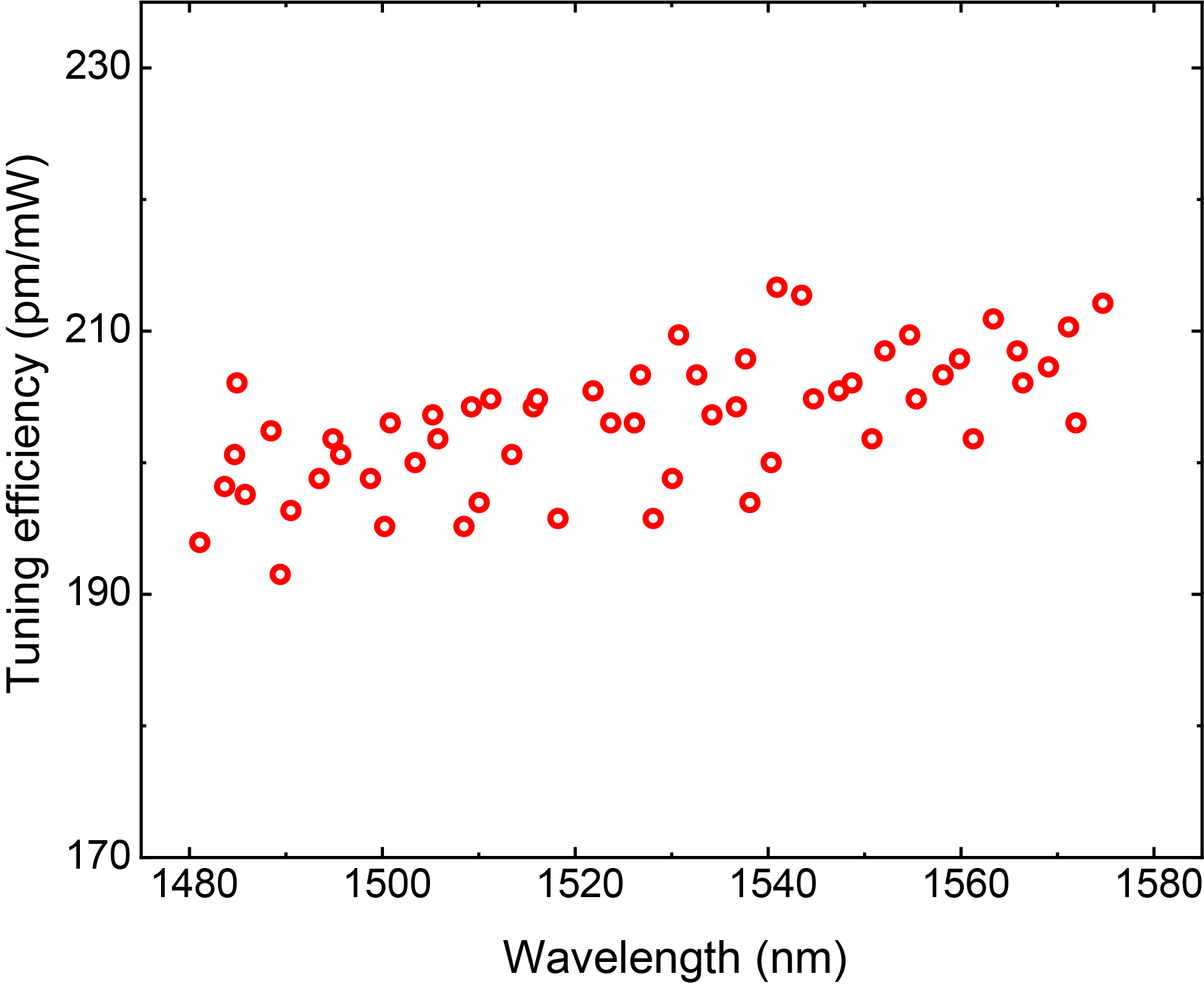


**Fig. S34** **|** Tuning efficiencies of the resonant modes of the chaotic cavity with respect to wavelength.

**References**

1 Cao H, Wiersig J. Dielectric microcavities: Model systems for wave chaos and non-Hermitian physics. *Rev Mod Phys* 2015; **87**: 61–111.

2 Oliver J, Lee W-B, Lee H-N. Filters with random transmittance for improving resolution in filter-array-based spectrometers. *Opt Express, OE* 2013; **21**: 3969–3989.

3 Zhang Z, Li Y, Wang Y, Yu Z, Sun X, Tsang HK. Compact High Resolution Speckle Spectrometer by Using Linear Coherent Integrated Network on Silicon Nitride Platform at 776 nm. *Laser & Photonics Reviews* 2021; **15**: 2100039.

4 Yao C, Chen M, Yan T, Ming L, Cheng Q, Penty R. Broadband picometer-scale resolution on-chip spectrometer with reconfigurable photonics. *Light Sci Appl* 2023; **12**: 156.

5 Yao C, Xu K, Zhang W, Chen M, Cheng Q, Penty R. Integrated reconstructive spectrometer with programmable photonic circuits. *Nat Commun* 2023; **14**: 6376.

6 Edelman A. *Eigenvalues and Condition Numbers of Random Matrices*. 1989.

7 Yang Z, Albrow-Owen T, Cui H, Alexander-Webber J, Gu F, Wang X *et al.* Single-nanowire spectrometers. *Science* 2019; **365**: 1017–1020.

8 Yoon HH, Fernandez HA, Nigmatulin F, Cai W, Yang Z, Cui H *et al.* Miniaturized spectrometers with a tunable van der Waals junction. *Science* 2022; **378**: 296–299.

9 Hansen PC. *Discrete inverse problems: insight and algorithms*. Society for Industrial and Applied Mathematics: Philadelphia, 2010.

10 Xu H, Qin Y, Hu G, Tsang HK. Integrated Single-Resonator Spectrometer beyond the Free-Spectral-Range Limit. *ACS Photonics* 2023; **10**: 654–666.

11 Sun C, Chen Z, Ye Y, Lei K, Ma H, Wei M *et al.* Scalable On-Chip Microdisk Resonator Spectrometer. *Laser & Photonics Reviews* 2023; **17**: 2200792.

12 Redding B, Liew SF, Sarma R, Cao H. Compact spectrometer based on a disordered photonic chip. *Nature Photon* 2013; **7**: 746–751.

13 Zheng S, Cai H, Song J, Zou J, Liu PY, Lin Z *et al.* A Single-Chip Integrated Spectrometer via Tunable Microring Resonator Array. *IEEE Photonics Journal* 2019; **11**: 1–9.

14 Liu T, Fiore A. Designing open channels in random scattering media for on-chip spectrometers. *Optica* 2020; **7**: 934.

15 Hadibrata W, Noh H, Wei H, Krishnaswamy S, Aydin K. Compact, High-resolution Inverse-Designed On-Chip Spectrometer Based on Tailored Disorder Modes. *Laser & Photonics Reviews* 2021; **15**: 2000556.

16 Li A, Fainman Y. On-chip spectrometers using stratified waveguide filters. *Nat Commun* 2021; **12**: 2704.

17 Yuan S, Naveh D, Watanabe K, Taniguchi T, Xia F. A wavelength-scale black phosphorus spectrometer. *Nat Photon* 2021; **15**: 601–607.

18 Redding B, Liew SF, Bromberg Y, Sarma R, Cao H. Evanescently coupled multimode spiral spectrometer. *Optica, OPTICA* 2016; **3**: 956–962.

19 Hartmann W, Varytis P, Gehring H, Walter N, Beutel F, Busch K *et al.* Waveguide-Integrated Broadband Spectrometer Based on Tailored Disorder. *Advanced Optical Materials* 2020; **8**: 1901602.

20 Hartmann W, Varytis P, Gehring H, Walter N, Beutel F, Busch K *et al.* Broadband Spectrometer with Single-Photon Sensitivity Exploiting Tailored Disorder. *Nano Lett* 2020; **20**: 2625–2631.

21 Cheng Z, Zhao Y, Zhang J, Zhou H, Gao D, Dong J *et al.* Generalized Modular Spectrometers Combining a Compact Nanobeam Microcavity and Computational Reconstruction. *ACS Photonics* 2022; **9**: 74–81.

22 Xu H, Qin Y, Hu G, Tsang HK. Cavity-enhanced scalable integrated temporal random-speckle spectrometry. *Optica, OPTICA* 2023; **10**: 1177–1188.

23 Zhang J, Cheng Z, Dong J, Zhang X. Cascaded nanobeam spectrometer with high resolution and scalability. *Optica, OPTICA* 2022; **9**: 517–521.

24 Zhang L, Zhang M, Chen T, Liu D, Hong S, Dai D. Ultrahigh-resolution on-chip spectrometer with silicon photonic resonators. *OEA* 2022; **5**: 210100–9.

25 Xu H, Qin Y, Hu G, Tsang HK. Breaking the resolution-bandwidth limit of chip-scale spectrometry by harnessing a dispersion-engineered photonic molecule. *Light Sci Appl* 2023; **12**: 64.

26 Zhao Y, Guo X, Xiang J, Zhao Z, Zhang Y, Xiao X *et al.* Miniaturized computational spectrometer based on two-photon absorption. *Optica, OPTICA* 2024; **11**: 399–402.

27 Wu G, Abid M, Zerara M, Cho J, Choi M, Ó Coileáin C *et al.* Miniaturized spectrometer with intrinsic long-term image memory. *Nat Commun* 2024; **15**: 676.

28 Uddin MG, Das S, Shafi AM, Wang L, Cui X, Nigmatulin F *et al.* Broadband miniaturized spectrometers with a van der Waals tunnel diode. *Nat Commun* 2024; **15**: 571.

29 Li A, Bao F, Wu Y, Wang C, He J, Pan S. An Inversely Designed Reconstructive Spectrometer on SiN Platform. *Laser & Photonics Reviews* 2024; **18**: 2301107.

30 Sun H, Qiao Q, Lee C, Zhou G. Chip-scale mid-infrared digitalized computational spectrometer powered by silicon photonics MEMS technology. *Photonics and Nanostructures - Fundamentals and Applications* 2024; **58**: 101231.

31 Darweesh R, Yadav RK, Adler E, Poplinger M, Levi A, Lee J-J *et al.* Nonlinear self-calibrated spectrometer with single GeSe-InSe heterojunction device. *Science Advances* 2024; **10**: eadn6028.

32 Zhang Z, Huang B, Zhang Z, Chen H. On-Chip Reconstructive Spectrometer Based on Parallel Cascaded Micro-Ring Resonators. *Applied Sciences* 2024; **14**: 4886.
